# Supplementary material for: Characterize direct protein interactions with enrichable, cleavable and latent bioreactive unnatural amino acids
Source: Nat Commun. 2024 Jun 18;15:5221. doi: 10.1038/s41467-024-49517-1 (PMC11189575; doi:10.1038/s41467-024-49517-1)
Supplement: Supplementary file 1 — Supplementary Information [file 41467_2024_49517_MOESM1_ESM.pdf]

Supplementary Figures

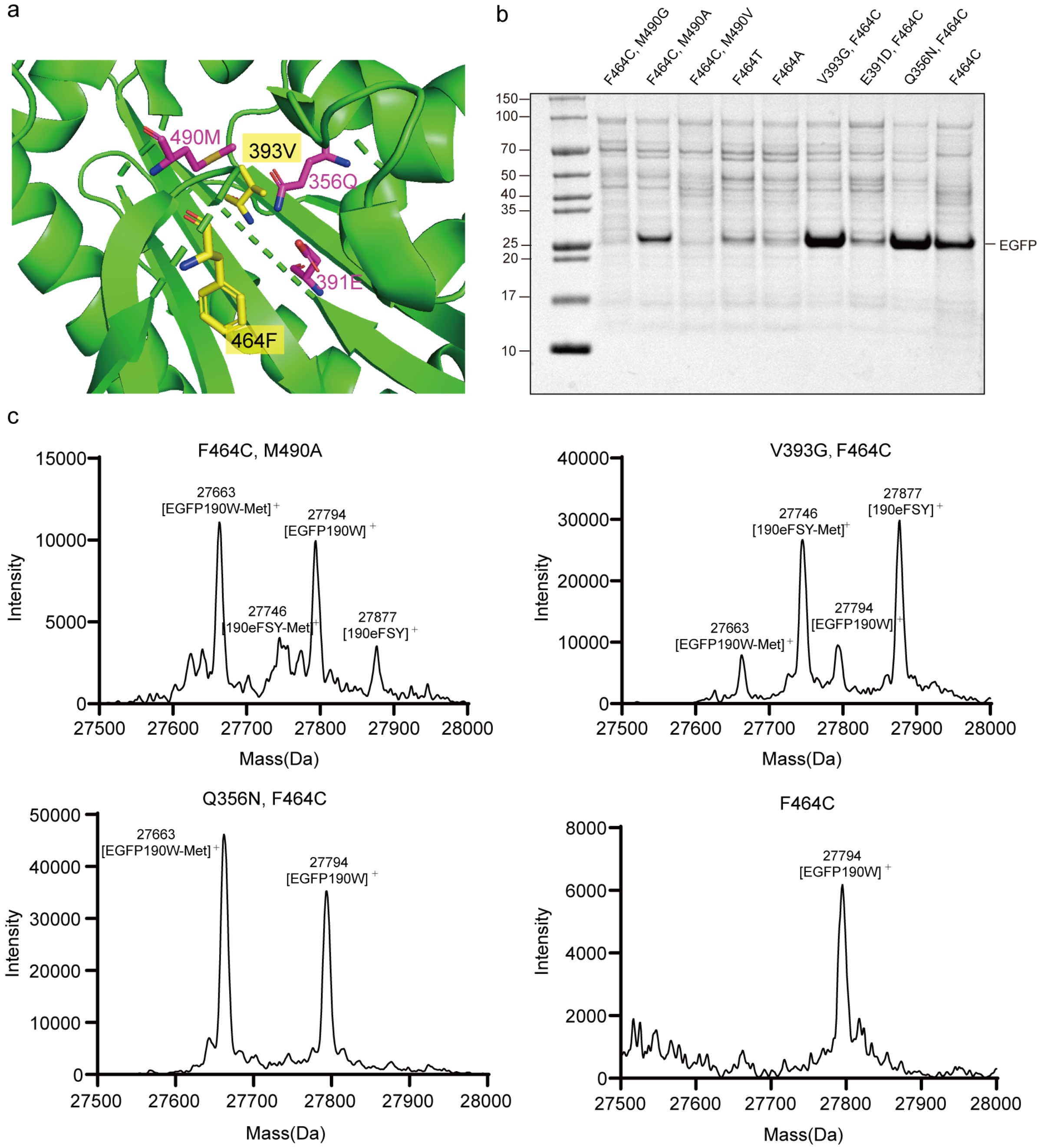

Supplementary Figure 1. Rational design of aminoacyl-tRNA synthetase. (a) Map mutation sites on protein structure of phenylalanyl tRNA synthetase (PDB ID: 5MGH). (b) SDS-PAGE gel of His-tag purified EGFP from *E. coli* cells co-expressing EGFP(190TAG) and chPheRS-1 mutants. Source data are provided as a Source Data file. (c) Mass spectra of intact EGFP purified from *E. coli* cells co-expressing EGFP(190TAG) and chPheRS-1 mutants.

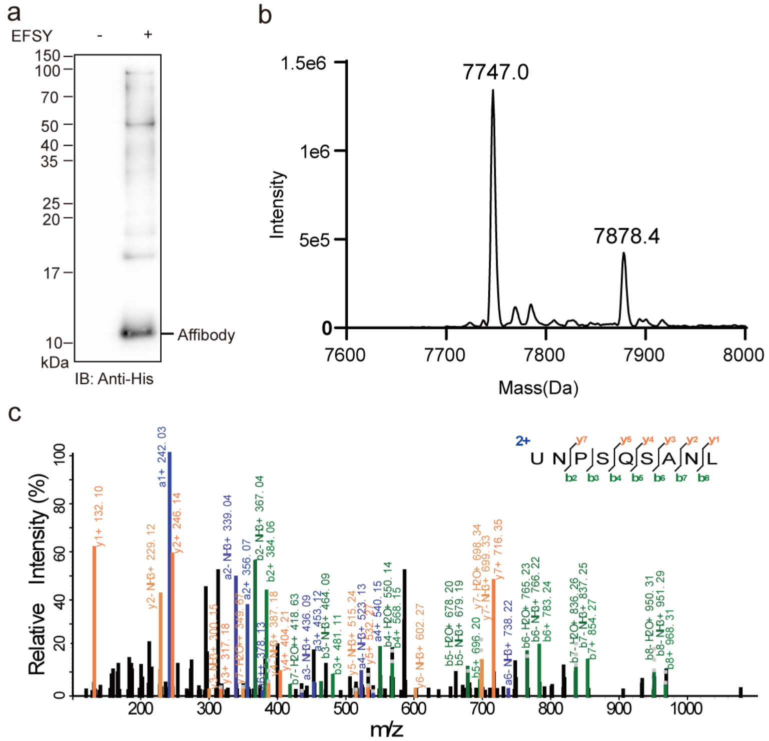

Supplementary Figure 2. Genetically encode eFSY into affibody in E. coli. (a) Western blot of His-tag purified affibody(E39eFSY). The experiment was repeated twice with similar results. Source data are provided as a Source Data file. (b) Intact protein mass analysis of affibody(E39eFSY). (c) Tandem mass spectrum of eFSY incorporated peptide of affibody(E39eFSY).

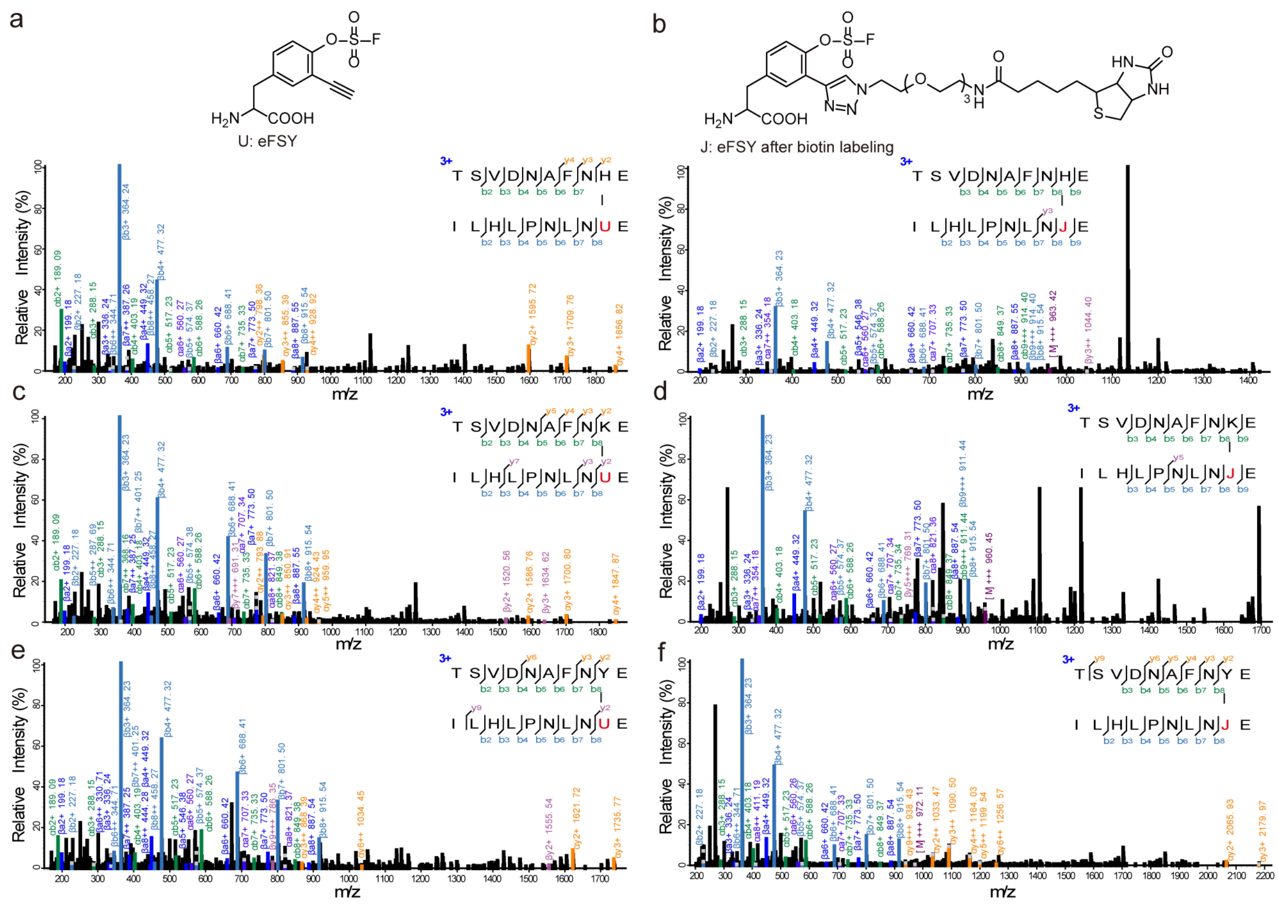

Supplementary Figure 3. Tandem mass spectra of cross-linked peptides and biotin labeled peptides. (a-b) Uaa-His cross-linking. (c-d) Uaa-Lys cross-linking. (e-f) Uaa-Tyr cross-linking.

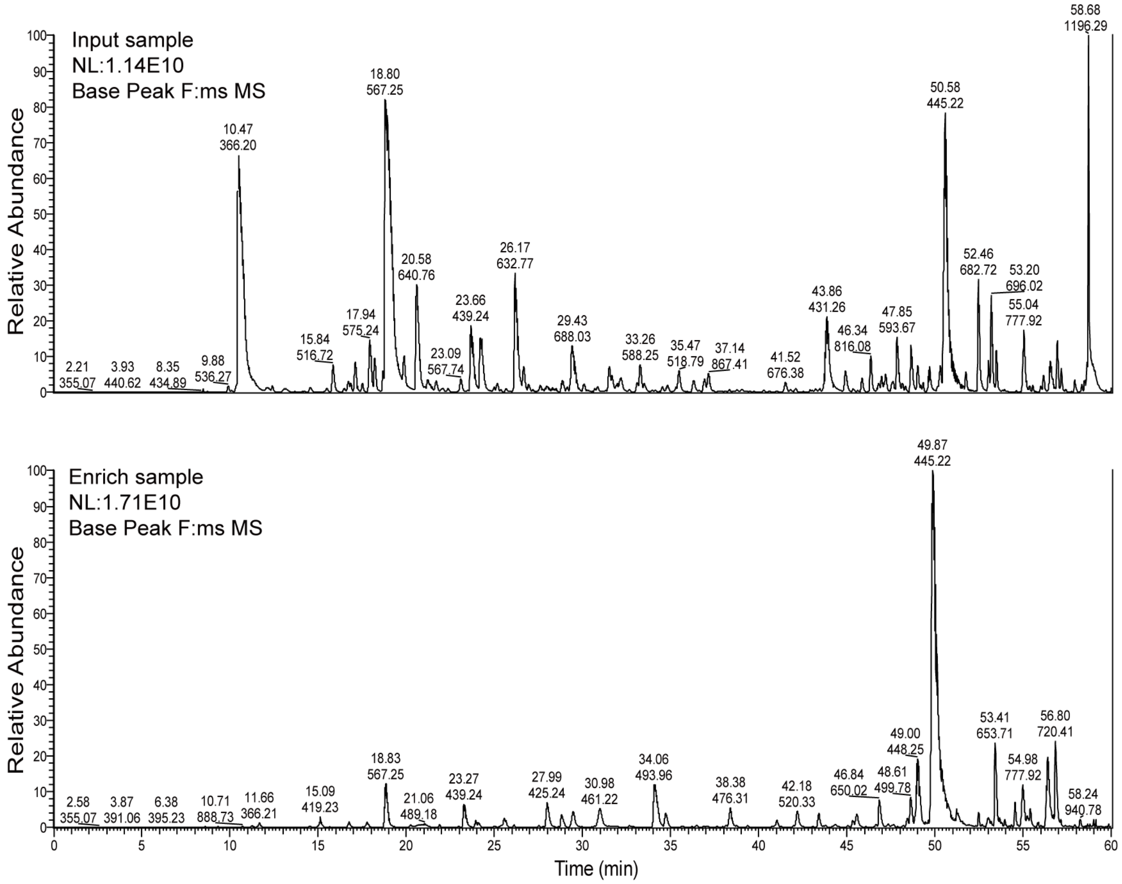

Supplementary Figure 4. Chromatography of input sample and enriched sample of affibody(A7H)/MBP-Z(E24eFSY) cross-linking.

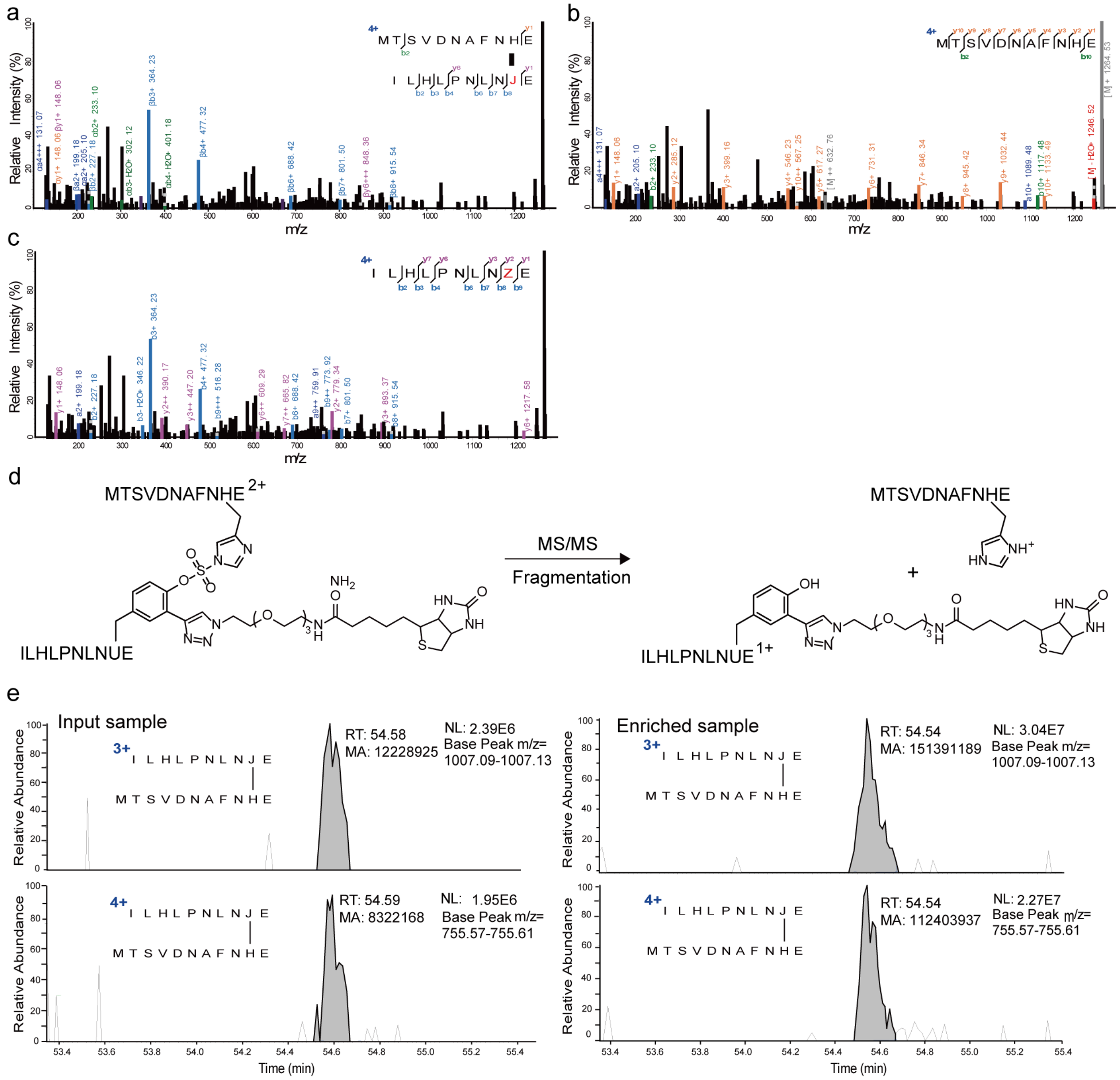

Supplementary Figure 5. Enriched cross-linked peptides of affibody(A7H)/MBP-Z(E24eFSY). (a) Tandem mass spectrum of cross-linked peptide MTSVDNAFNHE(10)-ILHLPNLNJE (9). (b) Labeling fragment ions of MTSVDNAFNHE after cross-linking bond fragmented. (c) labeling fragment ions of ILHLPNLNZE after cross-linking bond fragmented. (d) Fragmentation of cross-linking bond on cross-linked peptide MTSVDNAFNHE(10)-ILHLPNLNJE(9) (e) XICs of cross-linked peptides from input sample and enriched sample.

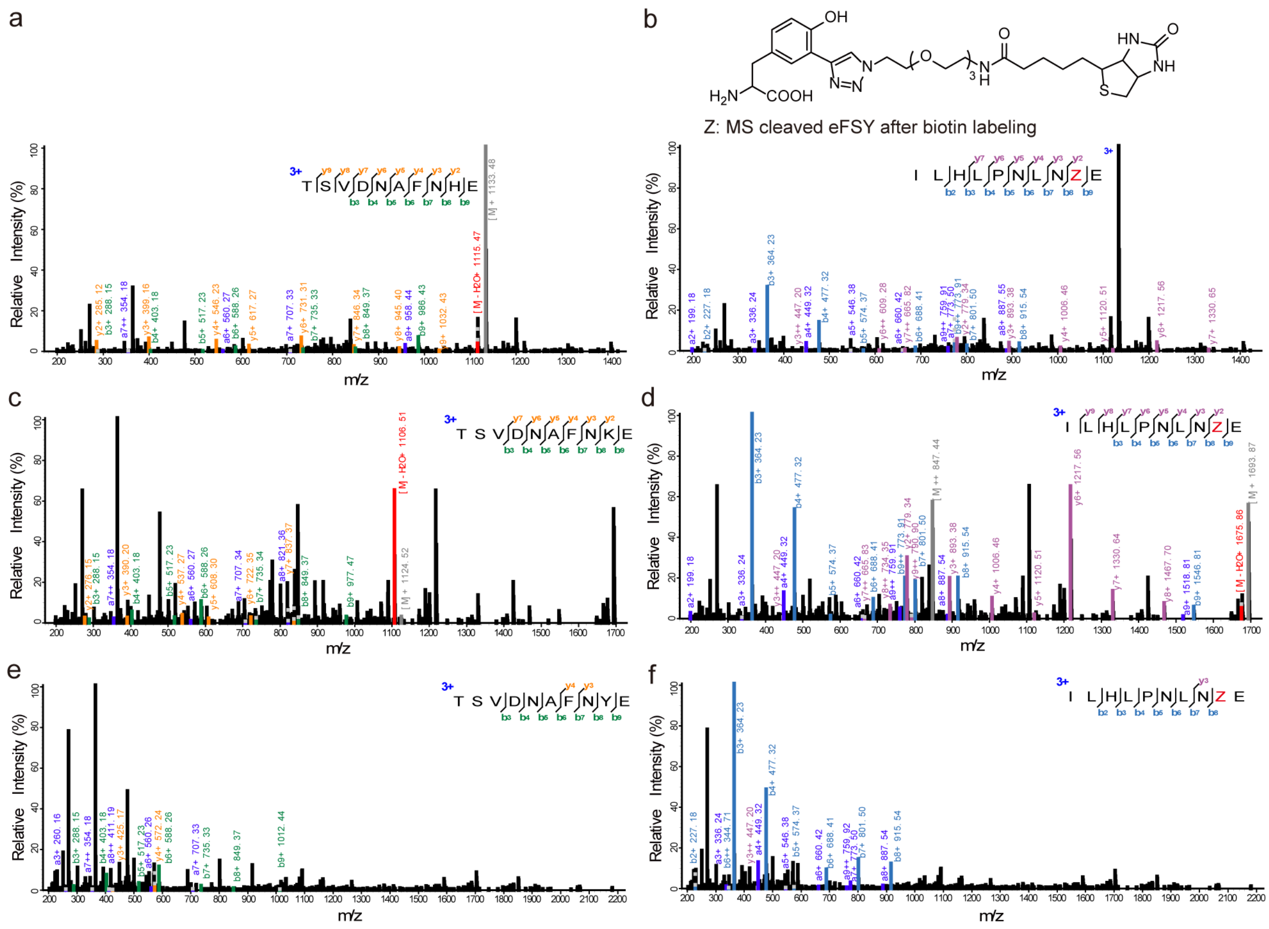

Supplementary Figure 6. Labeling internal fragment ions from cross-linking bond fragmentation on tandem mass spectra of biotinylated eFSY cross-linked peptides. (a-b) Uaa-His cross-linking. (c-d) Uaa-Lys cross-linking. (e-f) Uaa-Tyr cross-linking.

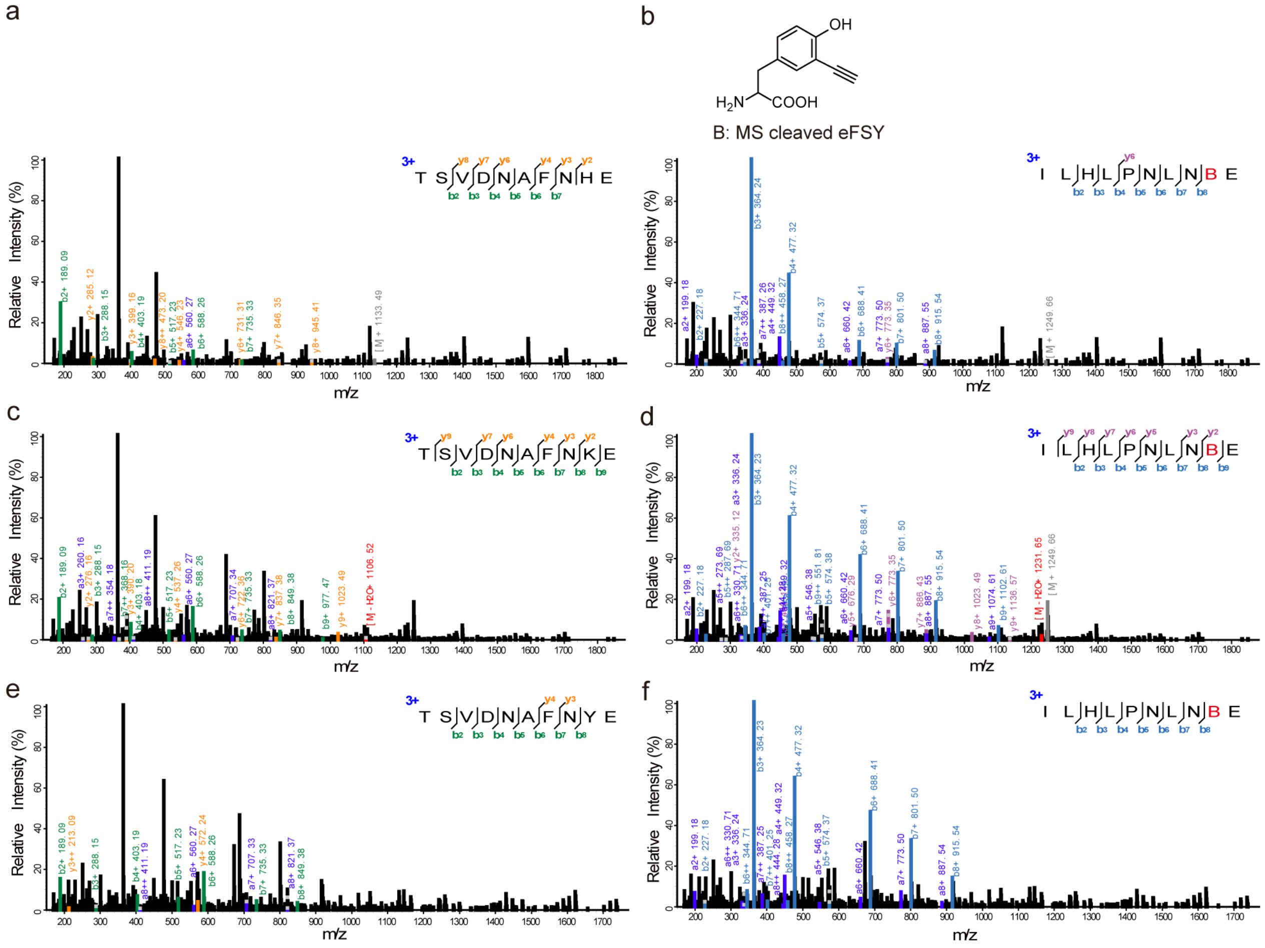

Supplementary Figure 7. Labeling internal fragment ions from cross-linking bond fragmentation on tandem mass spectra of unbiotinylated eFSY cross-linked peptides. (a-b) Uaa-His cross-linking. (c-d) Uaa-Lys cross-linking. (e-f) Uaa-Tyr cross-linking.

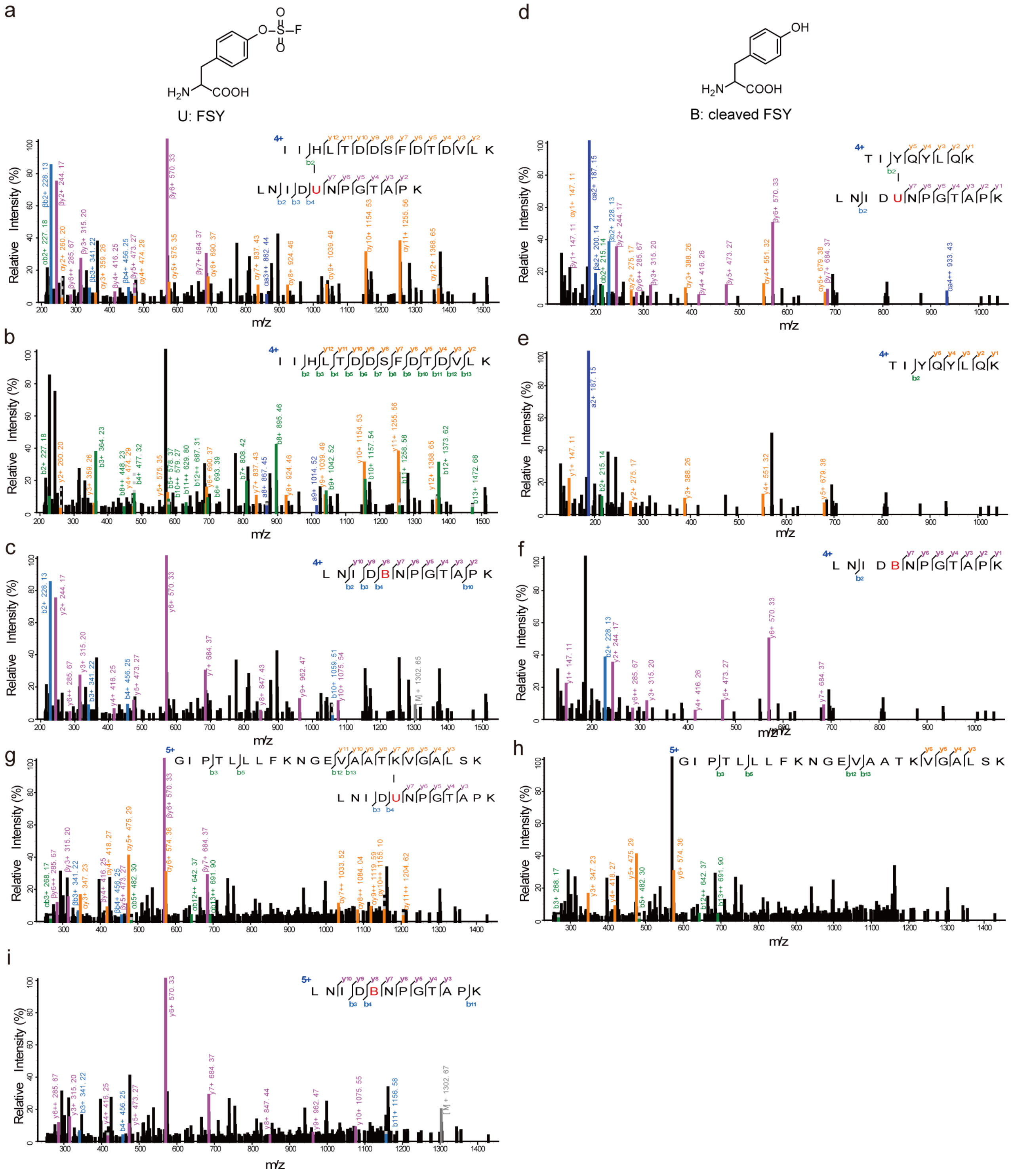

Supplementary Figure 8. Internal fragment ions on FSY mediated cross-linked peptides. (a-c) Uaa-His cross-linking. (d-f) Uaa-Tyr cross-linking. (g-i) Uaa-Lys cross-linking.

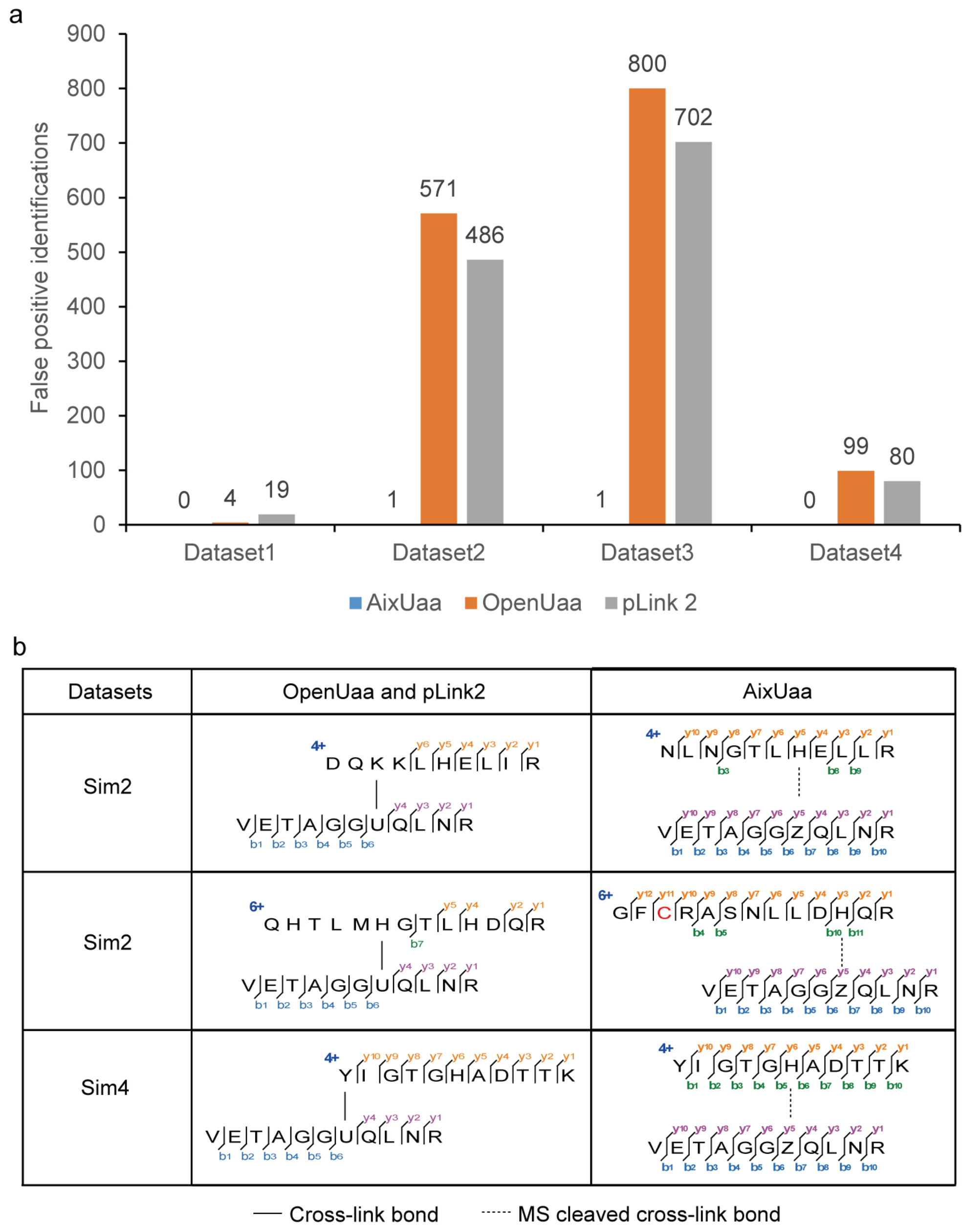

Supplementary Figure 9. Mis-assignments of peptides from different database search engines on simulated datasets. (a) OpenUaa and pLink2 report hundreds of mis-assignments (false positive) of peptides, especially in dataset Sim2 and dataset Sim3. (b) Examples of false positive identification from OpenUaa and pLink2.

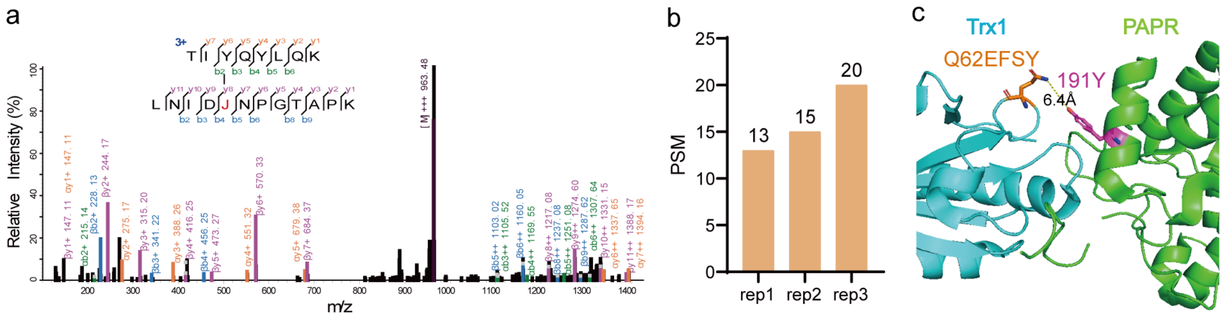

Supplementary Figure 10. Identified inter-protein cross-link between Trx1/PAPR. (a) Tandem mass spectra of inter-protein cross-linked peptide of Trx1/PAPR complex. (b) Peptide spectrum matches of cross-linked peptide TIYQYLQK(3)-LNIDJNPGTAPK(5). (c) Mapped cross-linking sites on protein structure of Trx1/PAPR (PDB ID 2O8V).

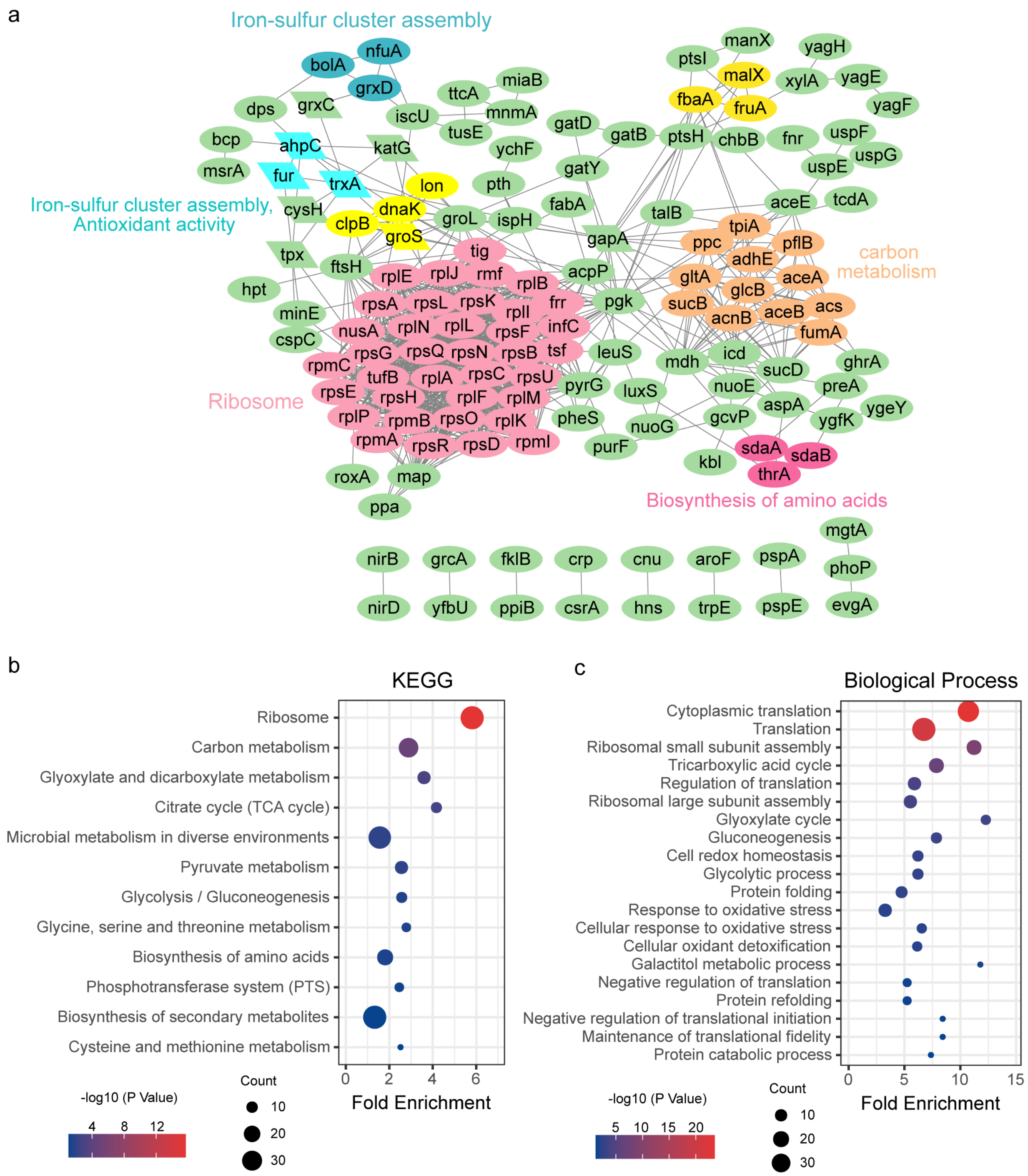

Supplementary Figure 11. eFSY captured direct interactome of Trx1. (a) STRING protein interaction network of Trx direct binding proteins. (b) KEGG pathway enrichment analysis of direct binding proteins of Trx1. (c) Gene ontology term analysis of direct binding proteins of Trx1.

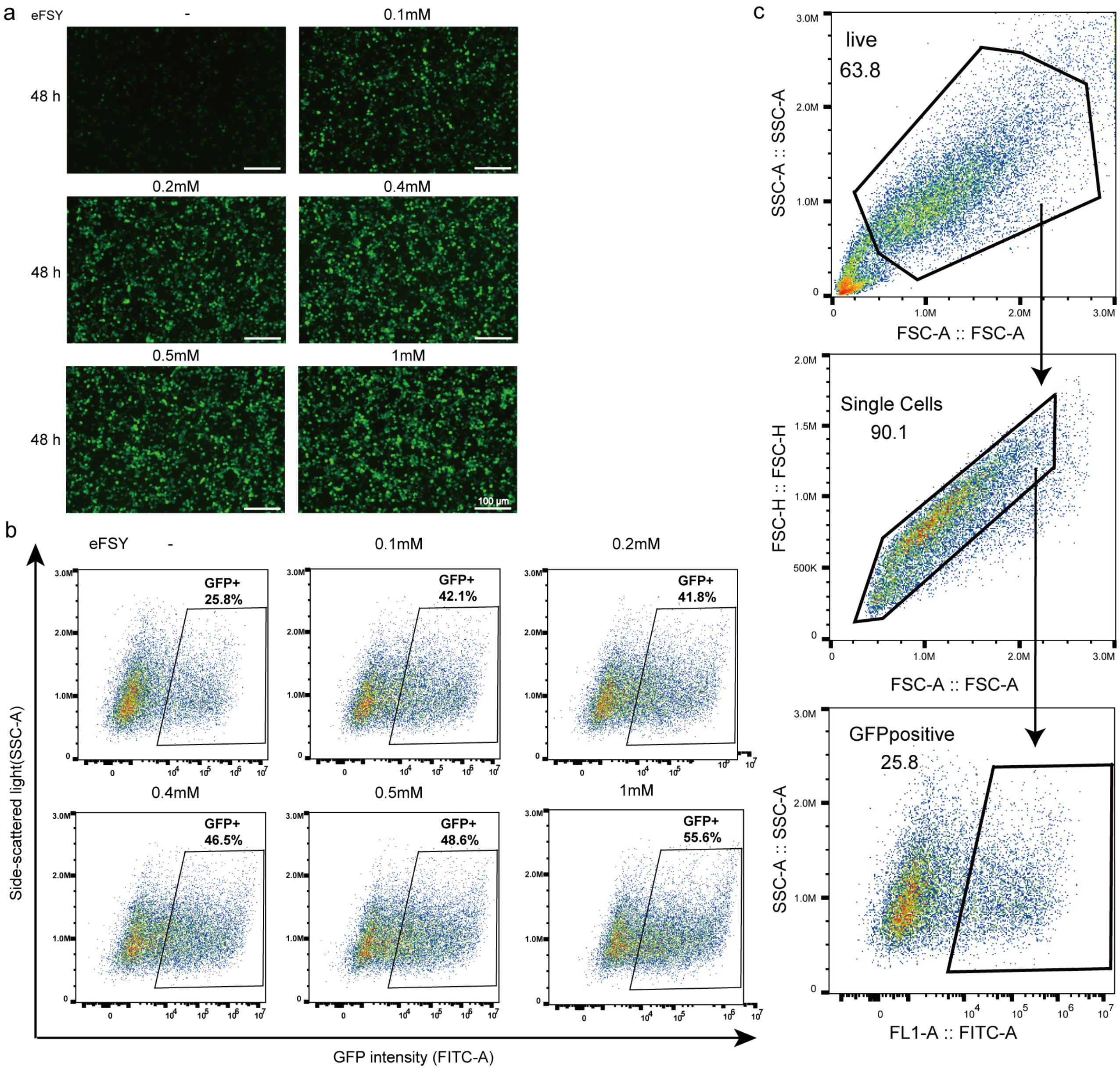

Supplementary Figure 12. Testing different eFSY concentration for its incorporation in HEK293T cells. (a) Fluorescence images of EGFP(Y151eFSY) with treatments of different concentration of eFSY. The experiment was repeated three times with similar results. (b) FACS analysis of eFSY incorporation into EGFP(Y151TAG) with treatments of different concentration of eFSY. The experiment was repeated three times with similar results. (c) Gating strategy in FlowJo. HEK 293T cells trasfected with EGFP were used to set appropriate forward scatter (FSC) and side scatter (SSC) gains. HEK 293T cells without transfection were used as negative control to set FITC gate. Each gate was highlighted by black boxes.

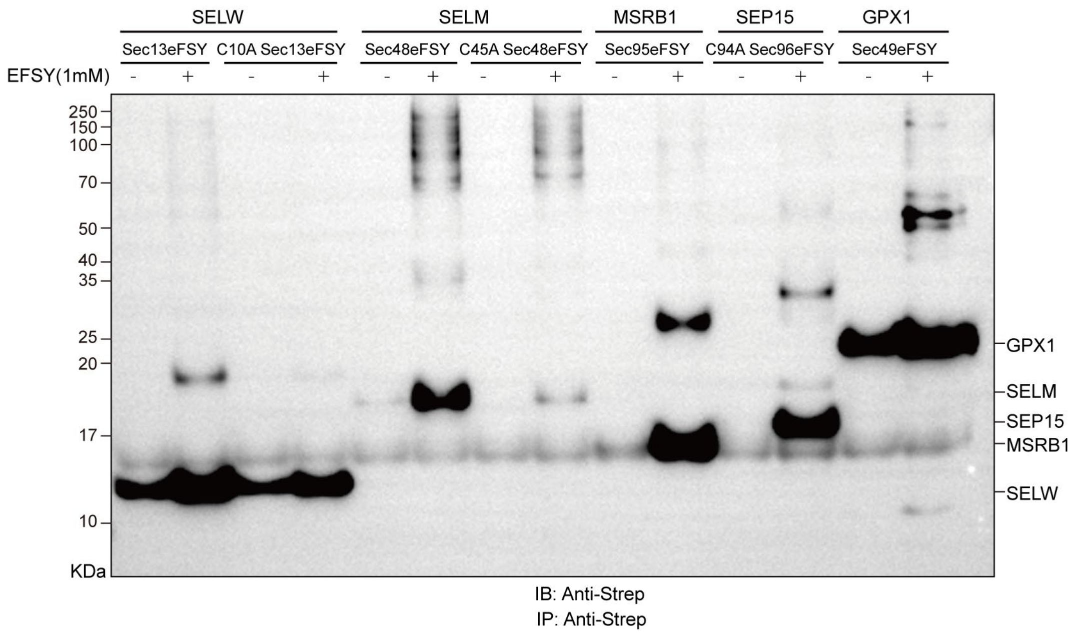

Supplementary Figure 13. Capturing endogenous interaction proteins of selenoproteins. Western blot analysis of strep-tag purified eFSY incorporated selenoproteins which were expressed in 293T cells, showing endogenous proteins cross-linked to selenoproteins. The experiment was repeated twice with similar results. Source data are provided as a Source Data file.

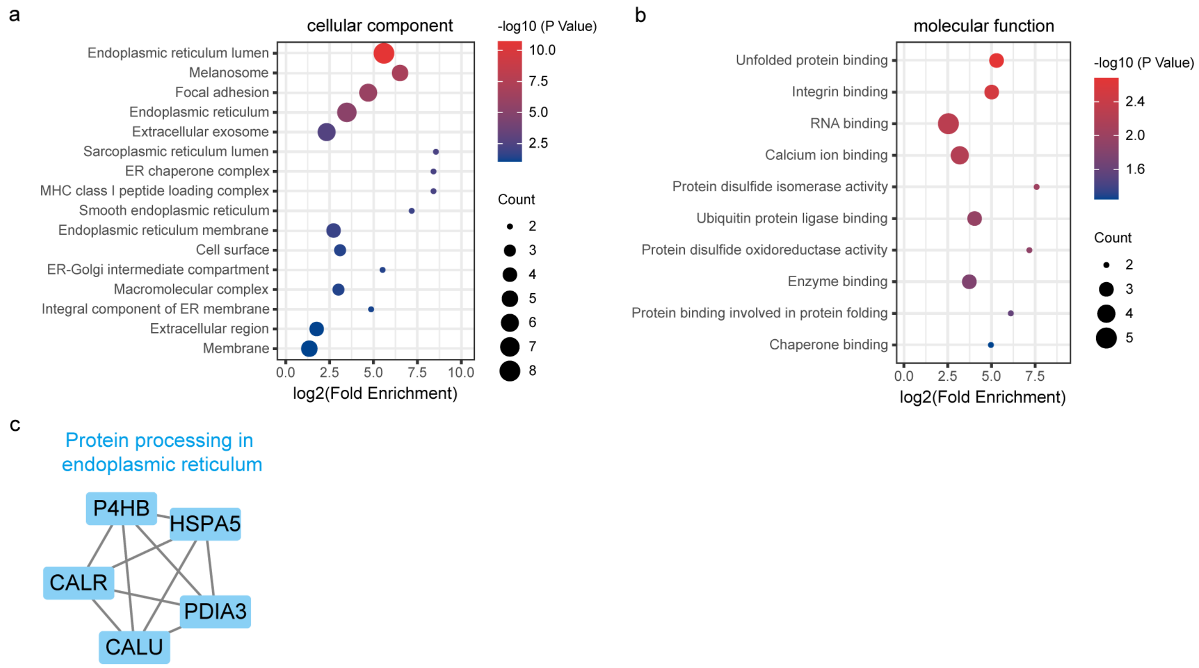

Supplementary Figure 14. Bioinformatics analysis on direct interacting proteins of SELM. (a-b) Gene ontology term analysis (c) STRING network analysis.

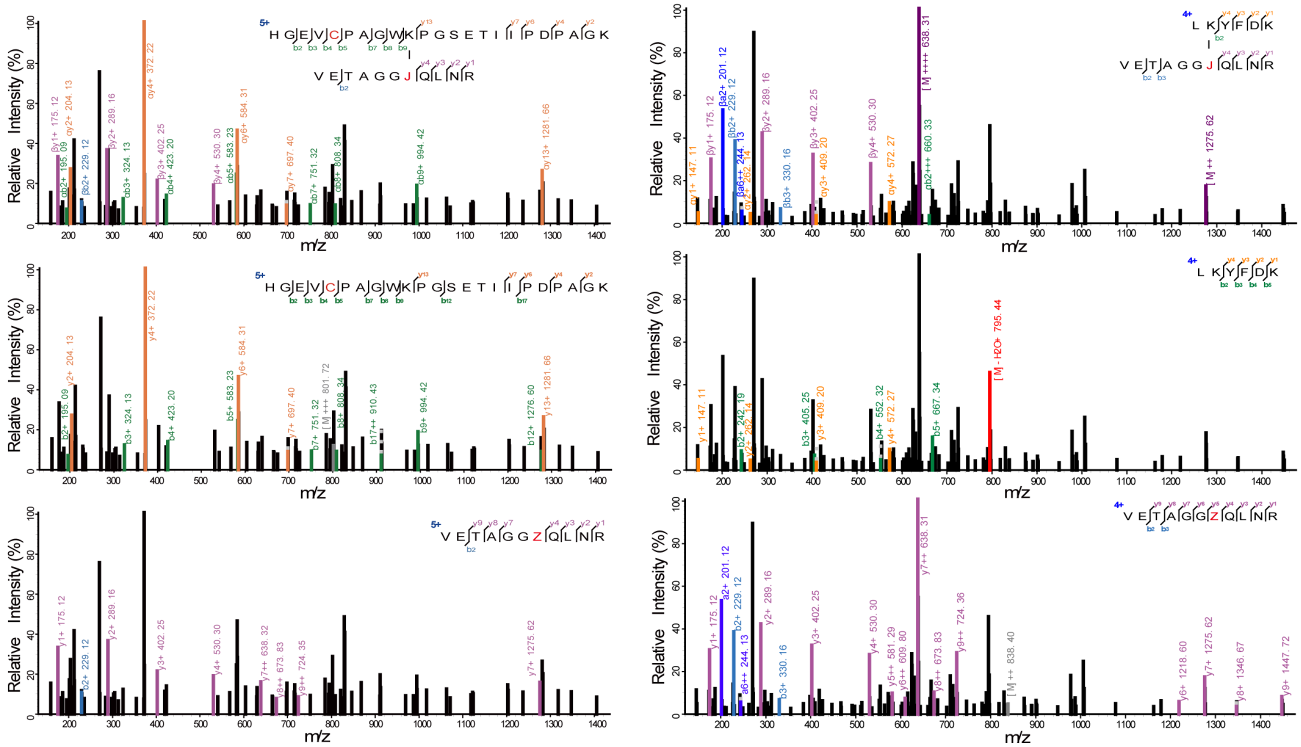

Supplementary Figure 15. Mass spectra of SELM and PRDX4 cross-linked peptides. Mass spectra of VETAGGJQLNR(7)-HGEVCPAGWKPGSETIIPDPAGK(10) and VETAGGJQLNR(7)-LKYFDK(2)

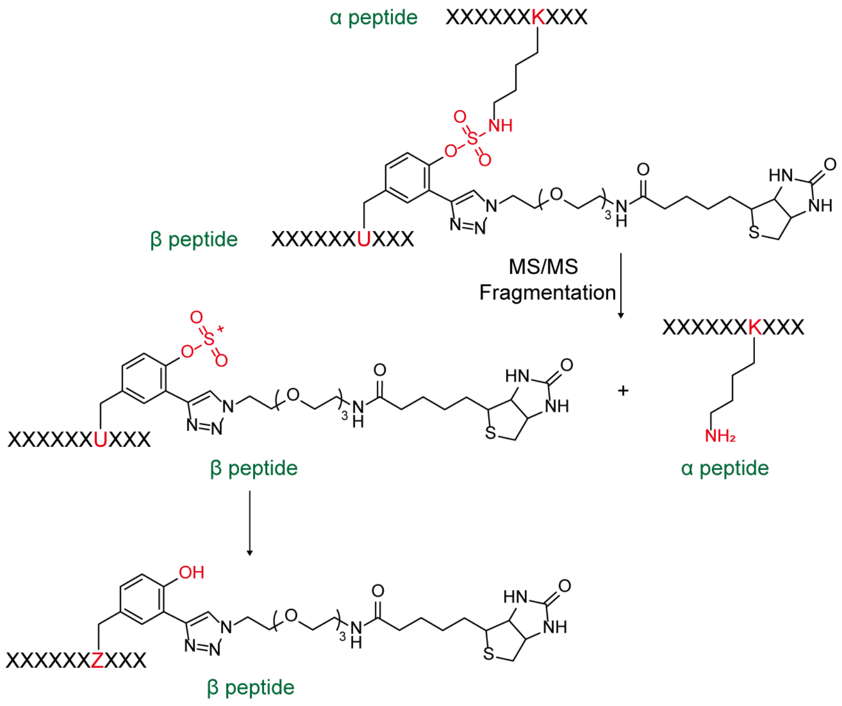

Supplementary Figure 16. Proposed fragmentation pathway of eFSY cross-linking. Sulfamate of eFSY-His and eFSY-Lys cross-linking can be protonated and cleaved, releasing two linear peptide**s.** Then linear β peptide ions containing eFSY residue are not stable and will be further cleaved.

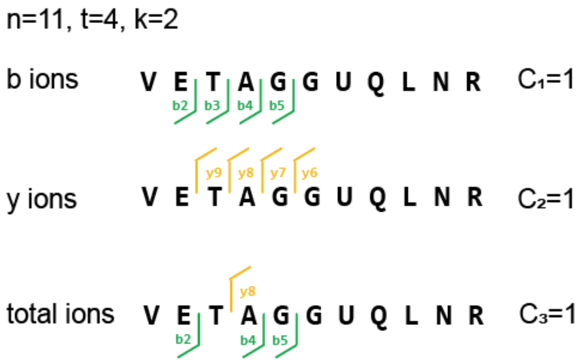

Supplementary Figure 17. Example for calculating C1, C2 and C3. For aa site 2 (E for VETAGGUQLNR), (1) there are at least 4 continuous b ions, so C1 = 1; (2) there are at least 4 continuous y ions, so C2 = 1; and (3) there are at least 4 continuous b or y ions, so C3 = 1; otherwise, C1, C2 and C3 = 0.

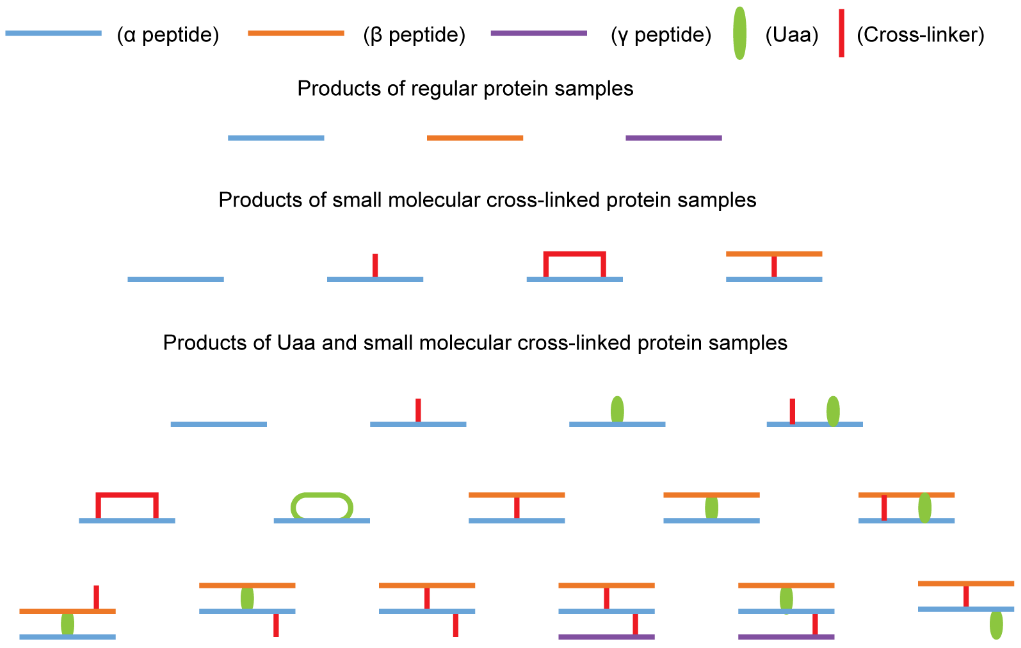

Supplementary Figure 18. Theoretical digested peptides of different protein samples. Regular protein samples only generate linear peptides after digestion. Small molecular cross-linked samples will generate linear peptides, mono-linked peptides, loop-linked peptides and cross-linked peptides. Uaa and small molecule cross-linker double cross-linked samples will generate 15 kinds of peptides.

Supplementary Tables

Supplementary Table 1. Rules for construction of simulated mass spectra dataset.

Dataset	#Total spectra	α peptide				β peptide
		Amino acid composition	Cross-linking site	Position of cross-linking site	Fragment ions	
1	1000	Contain only one Lys/His	Lys/His	On site 2-6 from C-terminus. Each position contains 200 mass spectra. Dataset 1, 2 and 3 have the same α peptide sequence.	Full set of b and y ions	Full set of b and y ions
2	1000				Full set of y ions and part of b ions	
3	1000				Full set of y ions and no b ions	
4	200	Contain at least one Lys/His and at least one Tyr			Full set of b and y ions	

Supplementary Notes

Supplementary Note 1. Synthesis of eFSY.

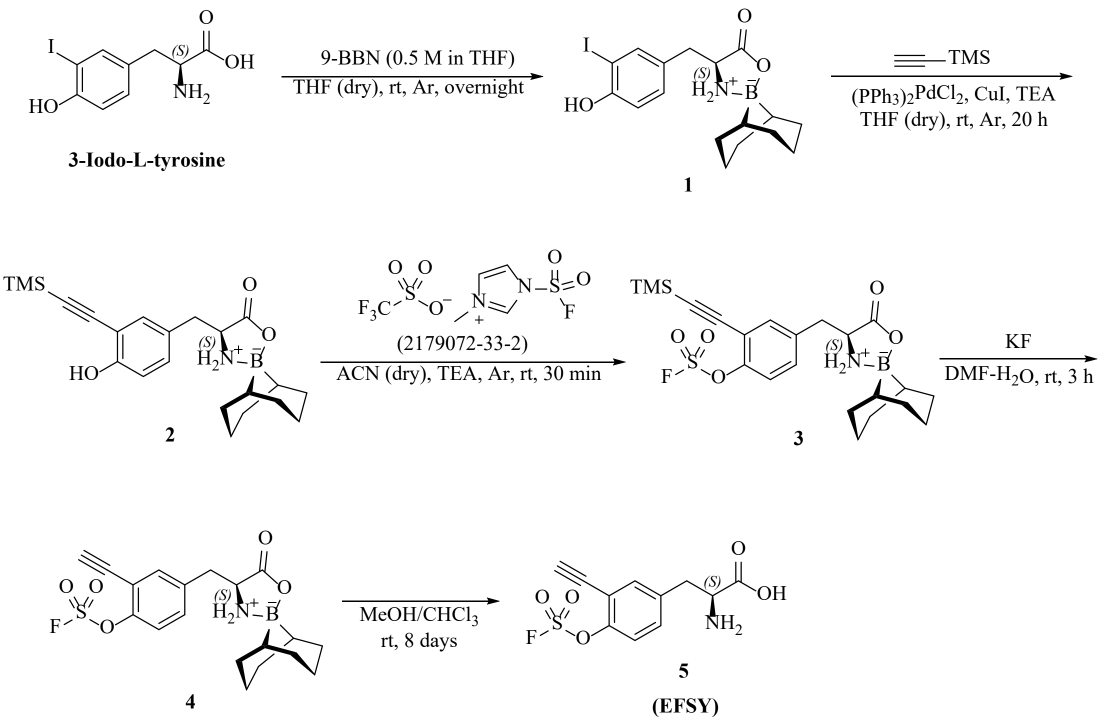

eFSY (**5**). 3-Iodo-L-tyrosine was protected with 9-BBN to give compound 1 that was coupled with ethynyltrimethylsilane under the Sonogashira cross-coupling conditions to yield compound 2. After an efficient sulfonyl fluorination under basic condition, TMS protective group was cleavaged to generate alkyne 4, which was stirred in MeOH/$CHCl_3$ to release free amino acid 5 (eFSY).

General information. All commercially available reagents and solvents (ACS grade) were purchased from commercial sources and used without further purification. Reactions were monitored by thin-layer chromatography (TLC) carried out on Merck silica gel 60 F-254 thin layer plates using UV light for visualization and an ethanolic solution of phosphomolybdic acid under heat or powdered iodine for developing. Flash column chromatography was generally performed on silica gel (200-300 mesh). LC-MS analyses were performed on the Agilent 1200 HPLC/MCD electrospray mass spectrometer in positive/negative ion mode. The scan range was 100−1000d. The yields refer to chromatographically

homogeneous materials. The ^{1}H and ^{13}C NMR spectra were recorded on a Bruker AV-500 or AV-400 spectrometer using $CDCl_3$ or DMSO-d_6 as solvent. The chemical shifts (δ) are reported in ppm and coupling constants (J) in Hz. The following abbreviations were used to explain the multiplicities: s = singlet, d = doublet, t = triplet. HRMS (High-resolution mass spectra) were conducted by a an Applied Biosystems Q-STAR Elite ESI-LC-MS/MS mass spectrometer under the condition of electrospray ionization (ESI).

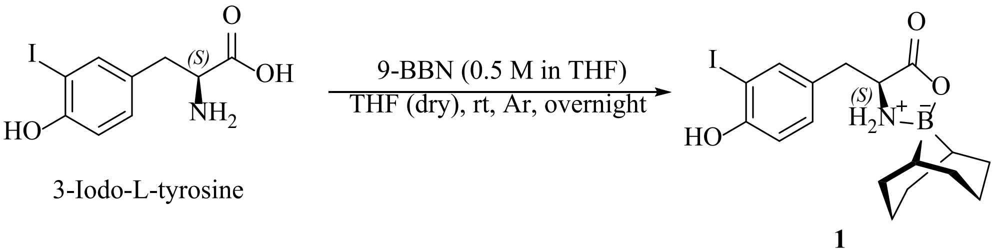

3-Iodo-L-tyrosine

1

Compound 1. 3-Iodo-L-tyrosine (10.0 g, 32.56 mmol, 1.00 equiv) was suspended in a solution of 9-BBN (0.5 M in THF, 100 mL, 50.00 mmol, 1.54 equiv), and was stirred under argon at ambient temperature overnight until all materials had dissolved. The product was precipitated by the addition of n-Hexane. After filtration, the resulting filter cake was washed with n-Hexane, then dried in vacuo to obtain compound **1** as white powder (13.0 g, Yield: 93.53%). ^{1}H NMR (500 MHz, DMSO-d_6) δ 10.09 (1H, br s, -O**H**), 7.65 (1H, d, J = 1.9 Hz, Ph**H**), 7.13 (1H, dd, J = 8.3, 1.9 Hz, Ph**H**), 6.80 (1H, d, J = 8.3 Hz, Ph**H**), 6.41 (1H, m, **-**N**H**$_2^+$**-**), 5.68 (1H, m, -N**H**$_2^+$-), 3.75 (1H, m, **-**C**H-**NH_2^+**-**), 3.03 (1H, dd, J = 14.6, 4.5 Hz, Ph**-CH**$_2$**-**), 2.78 (1H, dd, J = 14.6, 8.8 Hz, Ph**-CH**$_2$**-**), 1.86-1.18 (12H, m, 9-BBN (**-**C**H**$_2$**-**)), 0.45 (1H, br s, 9-BBN (**-**C**H-**)), 0.38 (1H, br s, 9-BBN (**-**C**H-**)). MS Calcd for $C_{17}H_{24}BINO_3$ 428.09 [M+H]$^+$, found 428.20 [M+H]$^+$, 469.10 [M+H+CH_3CN]$^+$, 855.02 [2M+H]$^+$.

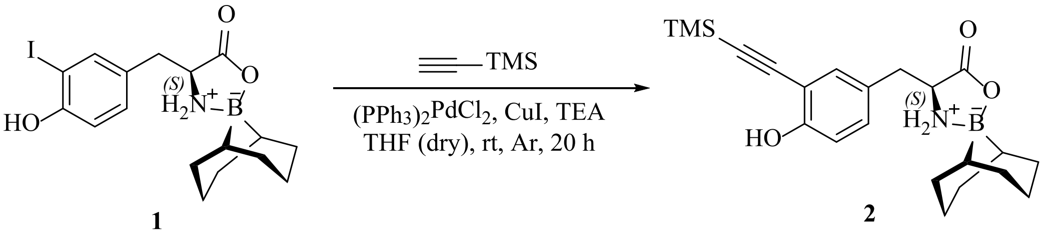

1 **2**

Compound 2. To a mixture of compound **1** (13.0 g, 30.42 mmol, 1.0 equiv), $(PPh_3)_2PdCl_2$ (512 mg, 0.73 mmol, 2.4 mol%), CuI (197 mg, 1.03 mmol, 3.4 mol%) and triethylamine (3.7 g, 36.50 mmol, 1.2 equiv) in 107 mL dry THF was addd ethynyltrimethylsilane (6.27 g, 63.88 mmol, 2.1 equiv) which was dissolved in 20 mL dry THF at ambient temperature, then the mixture was stirred at this temperature for 20 h. After the completion of the reaction, the supernatant was decanted and the insoluble was washed with THF, the combined THF solution was concentrated under reduced presssure. The desired product was isolated by silica column chromatography (Eluent: petroleum ether/ethyl acetate) to yield compound **2** as pail yellow powder (11.0 g, Yield: 90.91%). ^{1}H NMR (500 MHz, DMSO-d_6) δ 9.78 (1H, br s, -O**H**), 7.27 (1H, d, J = 2.0 Hz, Ph**H**), 7.13 (1H, dd, J = 8.4, 2.0 Hz, Ph**H**), 6.79 (1H, d, J = 8.4 Hz, Ph**H**), 6.38 (1H, m, -N**H**$_2^+$-), 5.68 (1H, m, -N**H**$_2^+$-), 3.74 (1H, m, -C**H**-NH_2^+-), 3.03 (1H, dd, J = 14.6, 4.4 Hz, Ph-**CH**$_2$-), 2.77 (1H, dd, J = 14.6, 8.9 Hz, Ph-**CH**$_2$-), 1.82-1.29 (12H, m, 9-BBN (-C**H**$_2$-)), 0.45 (1H, br s, 9-BBN (-C**H**-)), 0.41 (1H, br s, 9-BBN (-C**H**-)), 0.20 (9H, s, TMS). MS Calcd for $C_{22}H_{33}BNO_3Si$ 398.22 $[M+H]^+$, found 398.28 $[M+H]^+$, 439.38 $[M+H+CH_3CN]^+$, 795.30 $[2M+H]^+$.

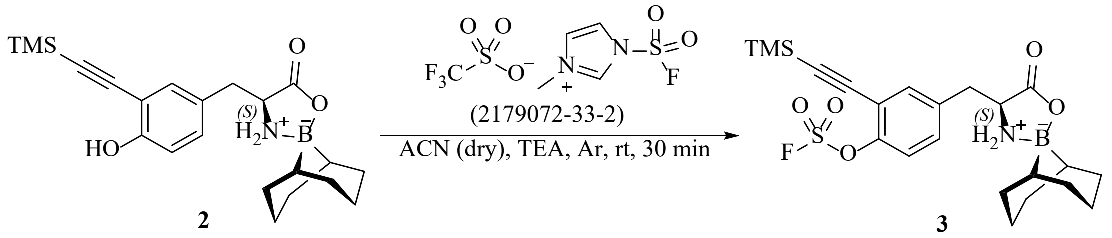

Compound 3. To a mixture of **2** (11.0 g, 27.68 mmol, 1.0 equiv) and TEA (4.2 g, 41.52 mmol, 1.5 equiv) in 100 mL dry acetonitrile was added sulfonyl fluorination reagent (CAS No: 2179072-33-2, 10.9 g, 33.22 mmol, 1.2 equiv) dissolved in 40 mL dry acetonitrile at ambient temperature. The reaction mixture was stirred at room temperature for 30 min. Solvent was removed under reduced pressure and the obtained residue was purified by silica column chromatography (Eluent: petroleum ether/ethyl acetate) to obtain the desired product as yellow solid (9.0 g, Yield: 67.82%). ^{1}H NMR (500 MHz, DMSO-d_6) δ 7.71 (1H, d, J = 1.9 Hz, Ph**H**), 7.62 (1H, d, J = 8.5 Hz, Ph**H**), 7.57 (1H, dd, J = 8.5, 1.9 Hz, Ph**H**), 6.39 (1H, m, -N**H**$_2^+$-), 5.93 (1H, m, -N**H**$_2^+$-), 3.94 (1H, m, -C**H**-NH_2^+-), 3.23 (1H,

dd, J = 14.7, 4.3 Hz, Ph-**C**H_2-), 2.92 (1H, dd, J = 14.7, 9.5 Hz, Ph-**C**H_2-), 1.86-1.20 (12H, m, 9-BBN (-C**H_2**-)), 0.46 (2H, br s, 9-BBN (2×-C**H**-)), 0.24 (9H, s, TMS). HRMS Calcd for $C_{22}H_{32}BFNO_5SSi$ 480.1842 $[M+H]^+$, found 480.1845 $[M+H]^+$.

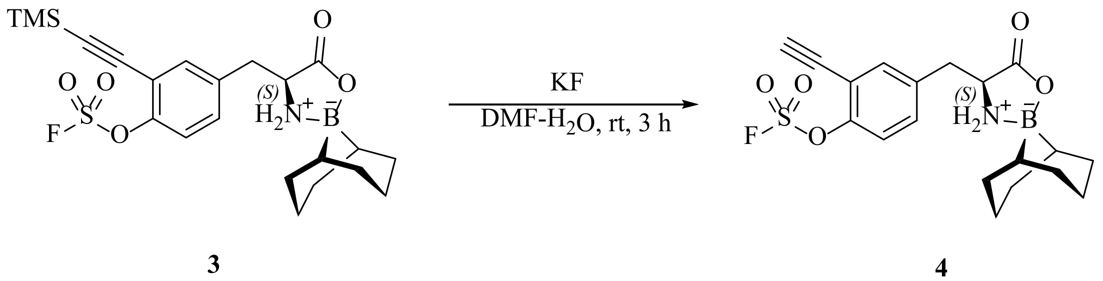

Compound 4. To a stirred solution of **3** (9.0 g, 18.77 mmol, 1.0 equiv) in 90 mL dry DMF was added 18 mL aqueous KF (2.5 g, 42.97 mmol, 2.3 equiv) at ambient temperature. The reaction mixture was stirred at room temperature for 3 h, then diluted with 225 mL 1 M $KHSO_4$ and extracted with ethyl acetate. The organic phase was washed with brine, dried over anhydrous Na_2SO_4 then concentrated to dryness. The residue was purified by silica column chromatography (Eluent: petroleum ether/ethyl acetate) to obtained the desired product as yellow solid. (7.2 g, Yield: 94.74%). ^{1}H NMR (500 MHz, DMSO-d_6) δ 7.74 (1H, d, J = 1.1 Hz, Ph**H**), 7.64 (1H, d, J = 8.5 Hz, Ph**H**), 7.59 (1H, dd, J = 8.5, 1.1 Hz, Ph**H**), 6.41 (1H, dd, J = 11.3, 7.4 Hz, -N**H_2**$^+$-), 5.95 (1H, m, -N**H_2**$^+$-), 4.70 (1H, s, Alkyne-**H**), 3.95 (1H, m, -C**H**-NH_2^+-), 3.24 (1H, dd, J = 14.7, 4.3 Hz, Ph-**C**H_2-), 2.95 (1H, dd, J = 14.7, 9.5 Hz, Ph-**C**H_2-), 1.84-1.30 (12H, m, 9-BBN (-C**H_2**-)), 0.47 (1H, br s, 9-BBN (-C**H**-)), 0.44 (1H, br s, 9-BBN (-C**H**-)). MS Calcd for $C_{19}H_{24}BFNO_5S$ 408.14 $[M+H]^+$, found 408.19 $[M+H]^+$, 449.15 $[M+H+CH_3CN]^+$, 518.19 $[2M+H]^+$.

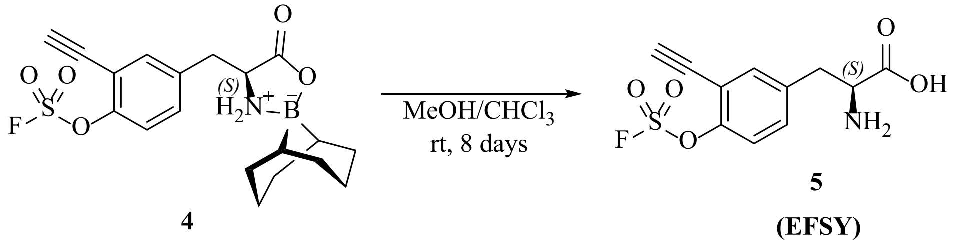

Compound 5 (eFSY). To a solution of **4** (4.0 g, 9.82 mmol) in 70 mL $CHCl_3$ was added 10 mL MeOH, the reaction mixture was stirred at room temperature for 8 days. 80 mL diethyl ether was added, the precipitate was filtered and washed well with diethyl ether, then dried in vacuo to obtain **5** (**eFSY**) as yellow powder (2.1 g, Yield: 75.00%). ^{1}H NMR (400 MHz, DMSO-d_6) δ 7.63 (2H, m, 2 × Ph**H**), 7.50 (1H, dd, J = 8.5, 1.6 Hz, Ph**H**), 4.69 (1H, s, Alkyne-**H**), 3.75 (1H, m, **-**C**H-**NH_2^+**-**), 3.16 (1H, dd, J = 14.3, 5.2 Hz, Ph**-CH**$_2$**-**), 3.00 (1H, dd, J = 14.3, 7.5 Hz, Ph**-CH**$_2$**-**). ^{13}C NMR (100 MHz, DMSO-d_6) δ 169.7, 148.7, 138.4, 135.6, 132.7, 121.9, 115.7, 87.8, 76.5, 54.0, 35.4. HRMS Calcd for $C_{11}H_{11}FNO_5S$ 288.0336 $[M+H]^+$, found 288.0353 $[M+H]^+$.

NMR and MS spectrums

^{1}H NMR of Compound 1.

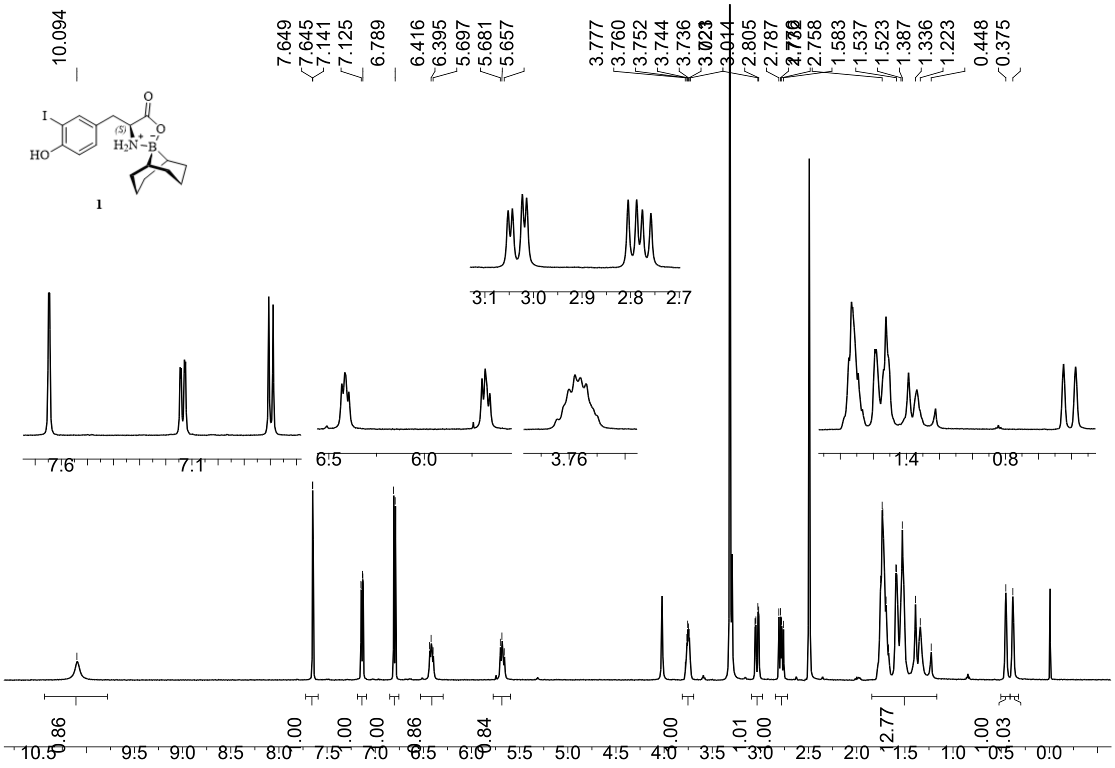

ESI-MS of Compound 1.

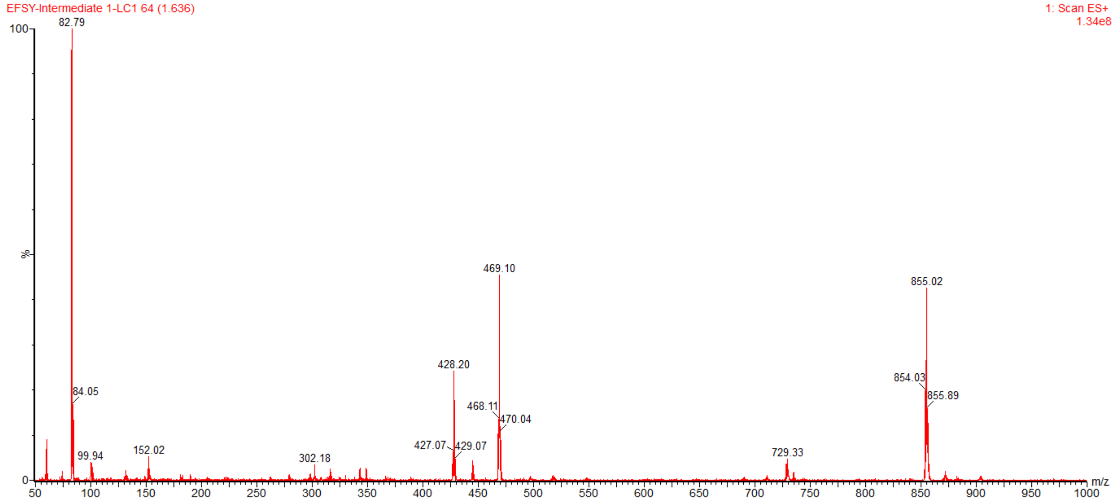

^{1}H NMR of Compound 2.

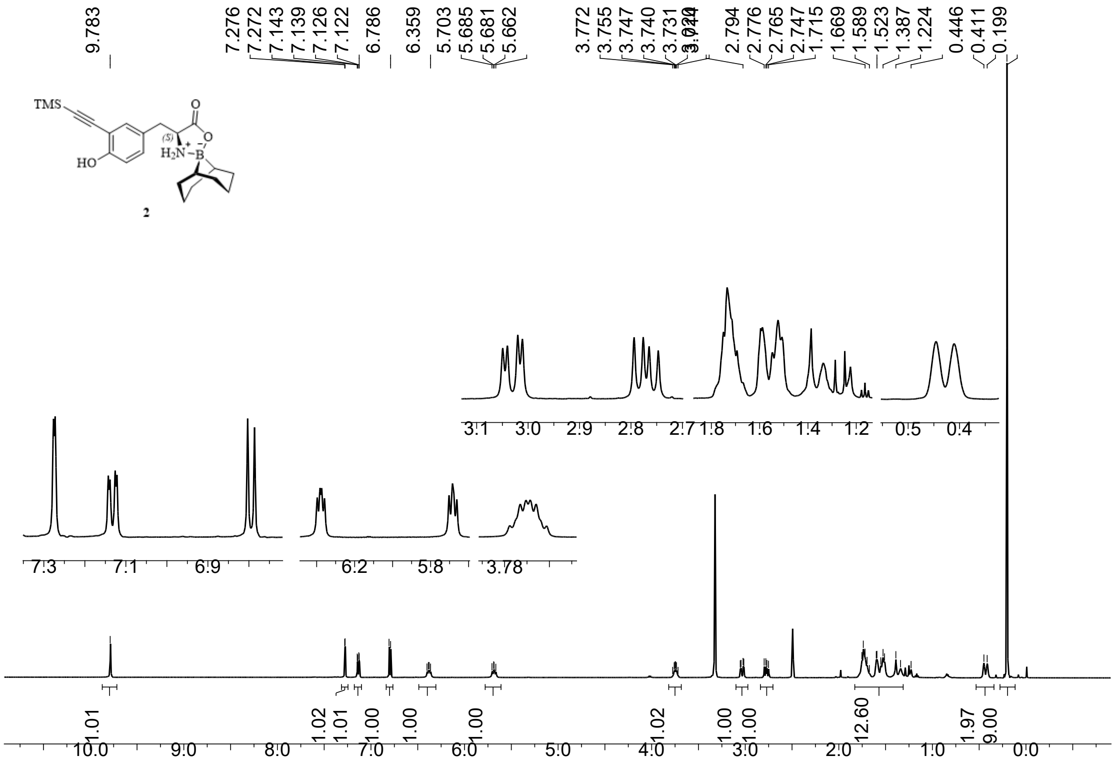

ESI-MS of Compound 2.

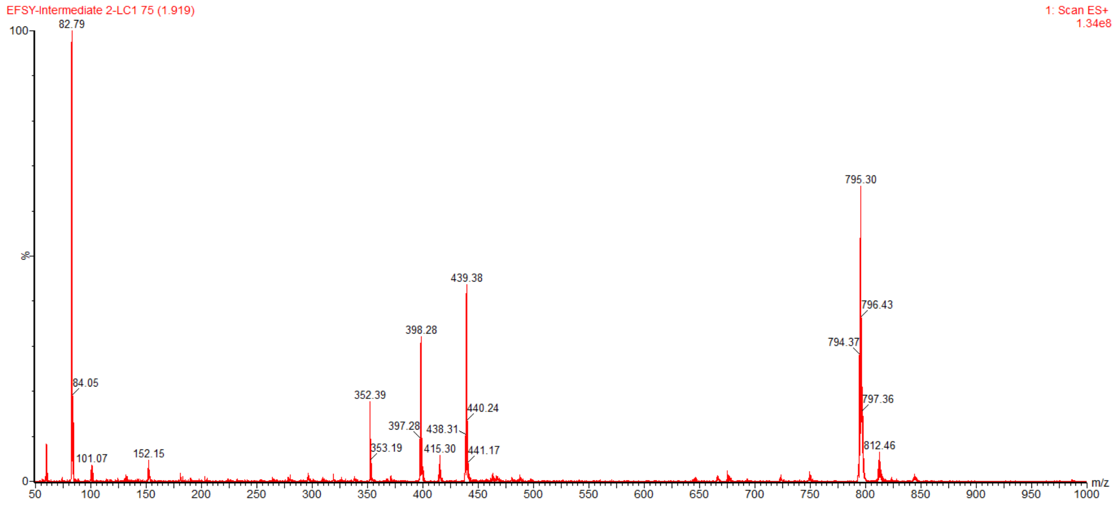

^{1}H NMR of Compound 3.

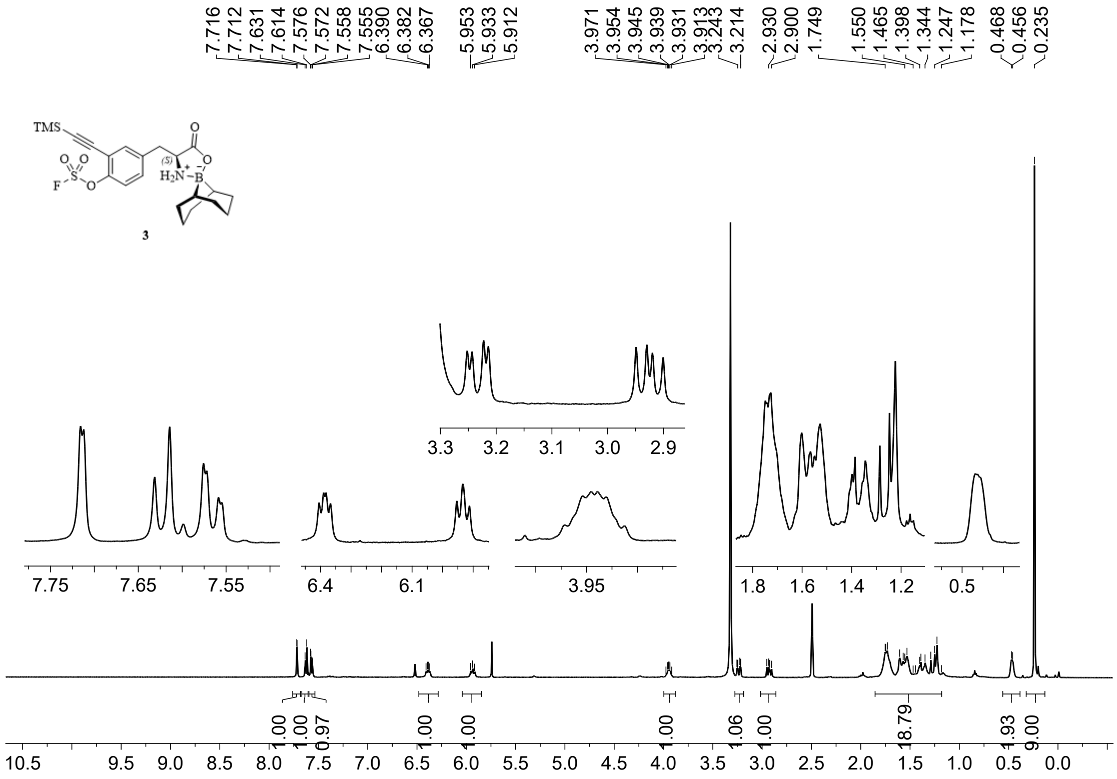

ESI-HRMS of Compound 3.

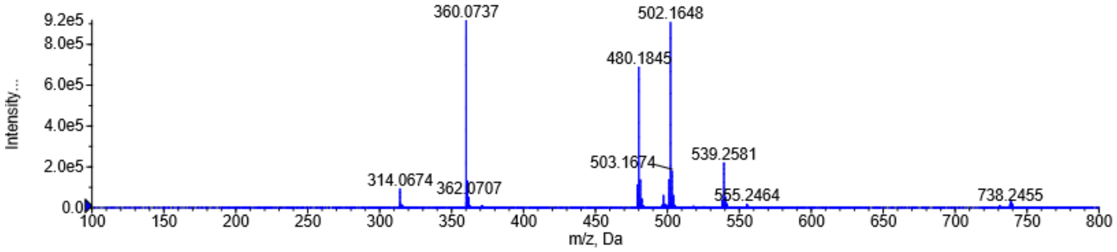

^{1}H NMR of Compound 4.

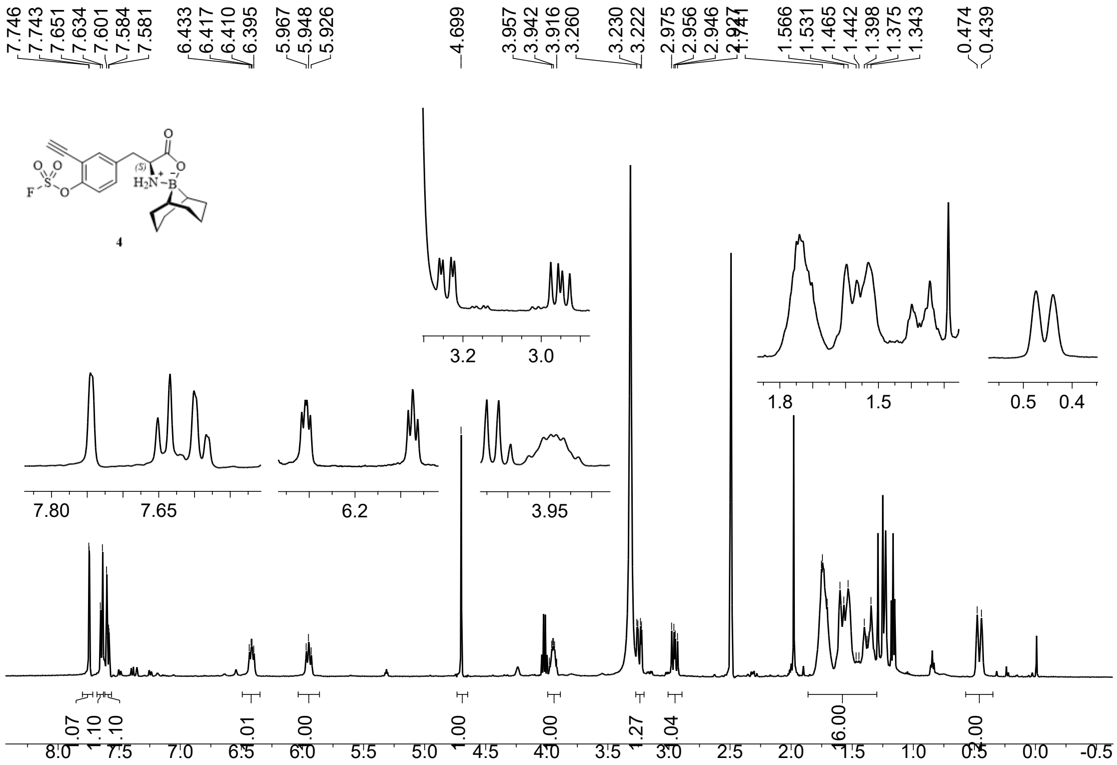

ESI-MS of Compound 4.

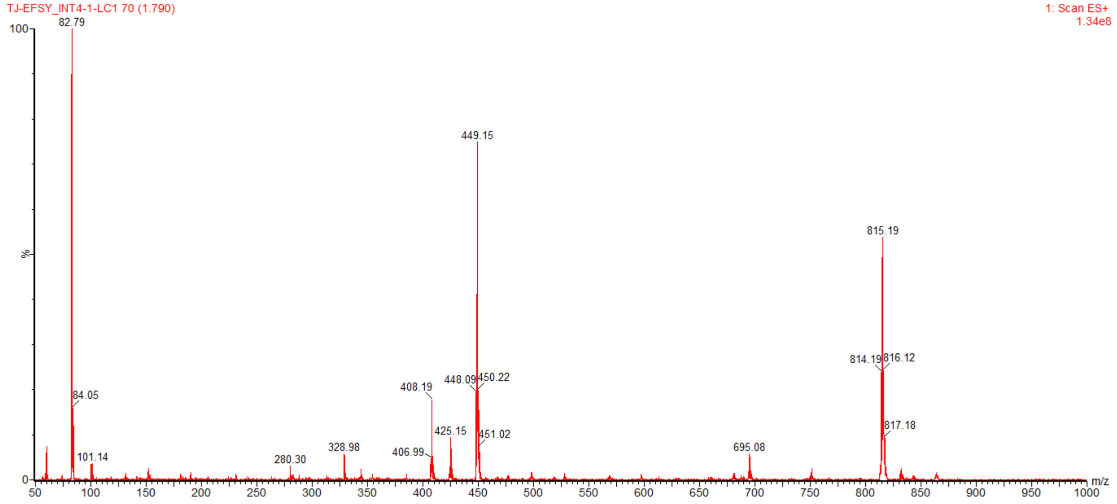

^{1}H NMR of eFSY.

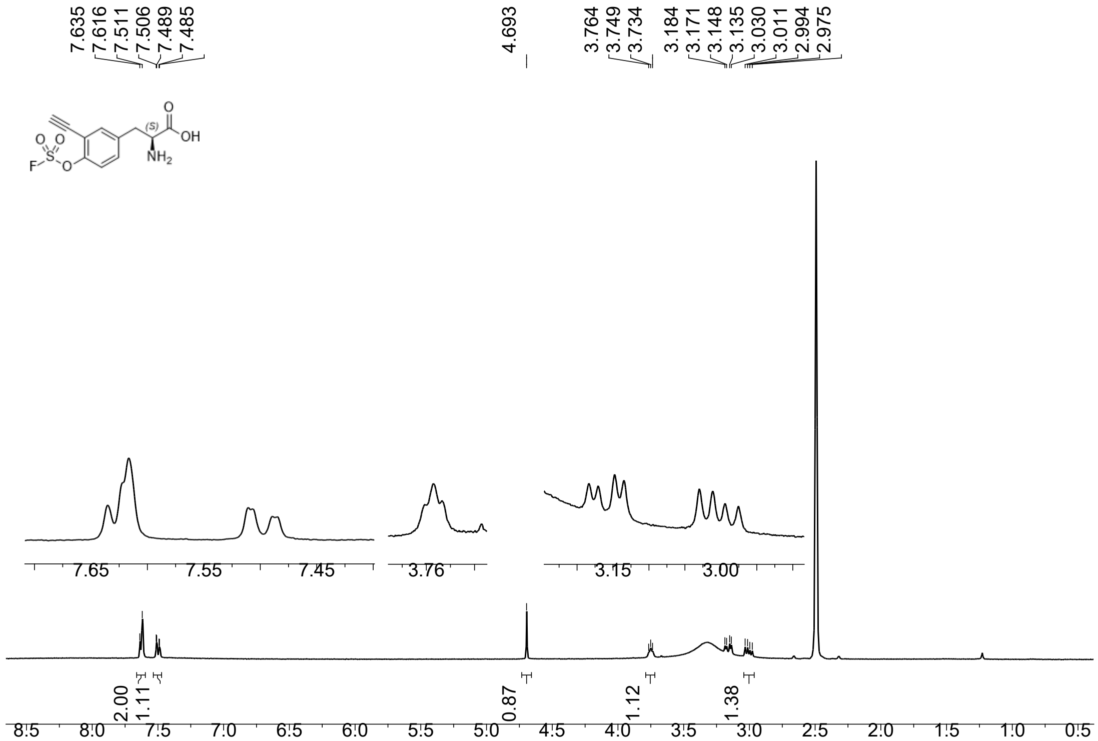

^{13}C NMR of eFSY.

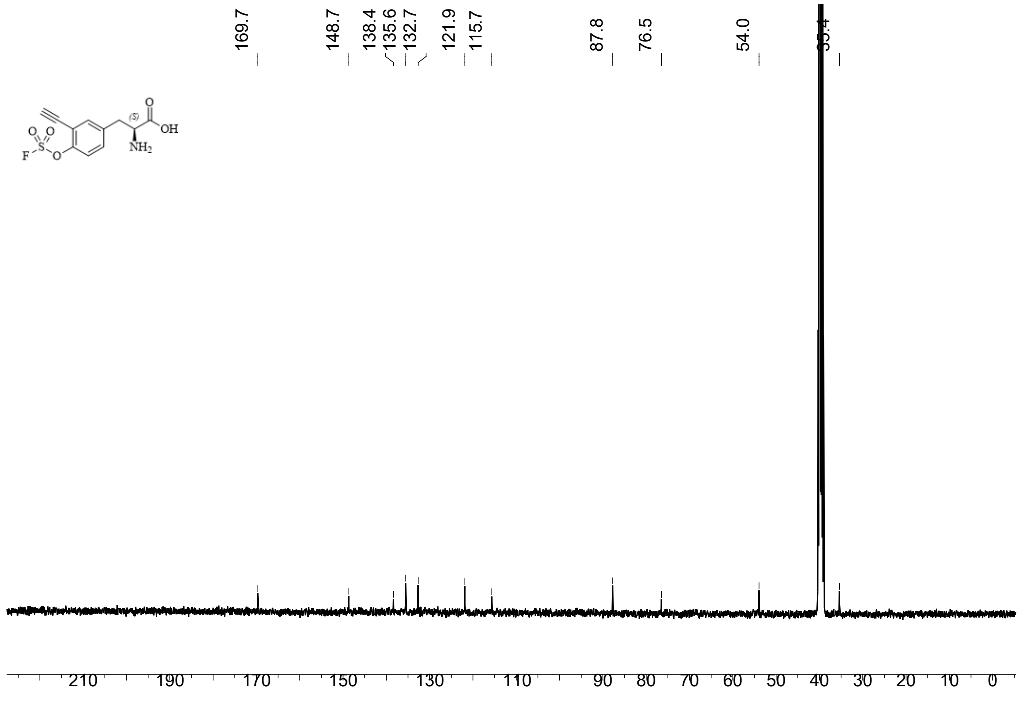

ESI-HRMS of eFSY.

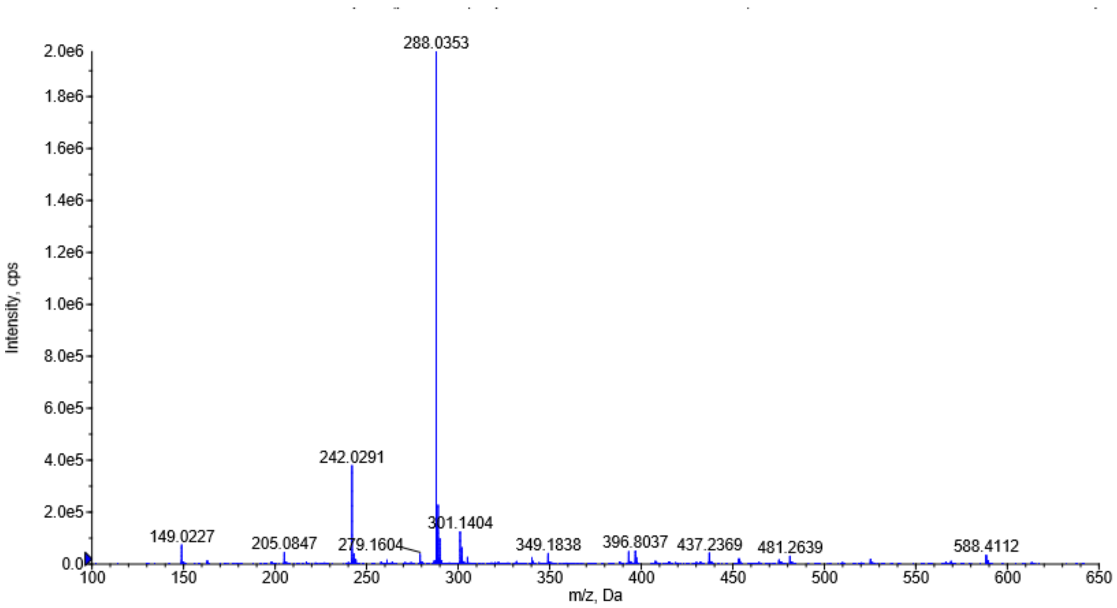

Supplementary Note 2. Development of AixUaa software

(1) Datasets for software testing

To test AixUaa, we constructed 4 simulated mass spectra datasets of eFSY-Lys/His cross-linking. Firstly, proteins of *Homo Sapiens* protein database (downloaded from uniport on October 16, 2023) were *in silico* digested into peptides with trypsin, and then modified regular peptides were generated with fixed modifications as Carbamidomethylation[C] and variable modifications as Oxidation[M]. β peptide is VETAGGUQLNR, α peptides were randomly chosen as shown in supplemental table 4. Secondly, precursor mass of cross-linked peptide is the sum of the mass of α peptide and β peptide, precursor charge state is randomly assigned within range of +2 to +6, then m/z of precursor is determined. Thirdly, all fragment ions of y^{1+}, y^{2+} were considered with their calculated m/z, while b^{1+}, b^{2+} were considered differently on different datasets as described in table 4.

(2) Design of AixUaa software

AixUaa contains four steps: (1) retrieving β peptides in open search mode; (2) creating database index for α peptides; (3) refined scoring on Amino Acid-dependent cleavable Uaa cross-linking peptides; (4) reranking.

Step1 retrieving β peptides in open search mode

Candidates of β peptides containing incorporated Uaa were generated by in silico digestion according to the given enzymes, modifications and protein sequences, then theoretical fragment ions were generated in the forms of cross-linked peptides or linear peptides, corresponding to the cross-linking with Histidine/Lysine or Tyrosine, separately. Each MS/MS spectrum was open-searched against candidates of β peptides. Spectrum can be retrieved only when satisfying following requirements: (1) containing at least n (default n=1) matched fragment ions (2) mass of candidate β peptide meeting the formula 1. m_β is the mass of the candidate β peptide, m_p is the experimental mass of the precursor

(converted to the singly charged state), m_{α_min} is the minimum mass of all candidate α peptides.

$$m_{\beta} \leq m_{p} - m_{\alpha\_min} \qquad \text{(Formula 1)}$$

Step2 creating database index for α peptides

Candidates of α peptides were generated from protein database based on user-defined enzymes and modifications. Theoretical precursor mass, modification, start position in the original protein sequence and protein name were recorded for further analysis. For each MS2 spectrum and the corresponding β peptide obtained in Step1, α peptide candidates were retrieved from the database index only when satisfying both requirements: (1) containing Histidine, Lysine or Tyrosine (2) mass of candidate α peptide meeting the formula 2. The MS2 spectra with paired α and β peptide candidates are preserved for fine-score.

$$m_{\alpha} = m_{p} - m_{\beta} \qquad \text{(Formula 2)}$$

Where m_α is the mass of candidate α peptide, m_β is the mass of the candidate β peptide, m_p is the experimental mass of the precursor (converted to the singly charged state).

Step3 refined scoring on AA-dependent cleavable Uaa cross-linking peptides

For the paired α and β peptide candidates obtained in step2, all theoretical b/y fragment ions were generated in the forms of crosslinked peptides or two individual linear peptides, corresponding to the cross-linking with Histidine/Lysine or Tyrosine, separately. Then the theoretical b/y fragment ions were fine-scored against the MS2 spectrum according to formula 3. Only the peptide pair with highest score is reported for each spectrum.

$$S_{refined} = S_{mat} + S_{con} \qquad \text{(Formula 3.1)}$$

$$S_{match} = \sum_{i} \log I_i \cos d_i \qquad \text{(Formula 3.2)}$$

$$S_{con} = \sum_{k=1}^{k=n-t} C_1 \sum_{j=k}^{j=k+t} \log I_{bj} \cos d_{bj} + C_2 \sum_{j=k}^{j=k+t} \log I_{yj} \cos d_{yj} + C_3 \sum_{j=k}^{j=k+t} \log I_j \cos d_j$$

(Formula 3.3)

Final match score ($S_{refined}$) was composed of product ion match score (S_{match}) and continuous score (S_{con}). The S_{match} considers the matched intensity (I) and mass tolerance of matched peak (d). The S_{con} consists of continuous score of b ions, y ions and total ions (b or y) as shown in formula 3.3, in which n is the peptide length, and t (default t=4) represents the minimum length of continuous ions. For a peptide with n amino acids, the S_{con} was calculated from the first amino acid to the (n-t) amino acid. For each site, C_1, C_2 or C_3 =1 when all theoretical ions from current site (k) to the following t ions (k+t) match with MS2 spectrum, otherwise C_1, C_2 or C_3=0 (Supplementary Fig. 17).

Step4 Reranking

Semi-supervised Lib-SVM framework is used to re-rank all PSMs and calculate FDR. The SVM model was trained with the following 8 features. In each iteration, the cross-linked peptides from target database within FDR≤5% are regarded as positive results and all cross-linked peptides from decoy database are regarded as negative results. After five iterations, all PSMs are ranked by SVM score and spectrum-level FDR is reported.

Features for SVM model:

(1) refined score

(2) delta refined score between rank first and rank second peptides pair

(3) The sum of the match score of α and β peptides

(4) α peptide match score

(5) α peptide continuous score

(6) α peptide matched ion ratio (the number of matched ions divided by total number of ions)

(7) α peptide matched intensity ratio (summed intensity of matched peaks divided by total intensity of peaks

(8) α peptide number of missed enzyme cleavage sites divided by logarithm of α peptide length

Supplementary Methods

Detect the in vivo crosslinking of GSTA and selenoproteins in mammalian cell

HEK 293T cells were cultured in 6 well-cell culture dishes until 80% cell confluency. The plasmids pNEU-eFSYRS and pRK5M with the GSTA or selenoproteins bearing the Amber stop codon were co-transfected. Six hours post transfection, the media were replaced with fresh DMEM media with 10% FBS in the presence or absence of 1 mM eFSY. After 48 hours, cells were collected, lysed and immunoprecipitated by 10ul prewashed His beads or Streptactin Beads 4FF. Beads were resuspended in 15ul 1×SDS loading and boiled at 95℃ for 5 minutes. The supernatant was analyzed by western blot.

Bioinformatics Analysis

KEGG pathway enrichment and Gene Ontology analysis were performed on DAVID.[1, 2] All identified interacting proteins of Trx or SELM were uploaded as input while using Escherichia coli str. K-12 substr. MG1655 or Homo sapiens as background separately. Results were visualized by Hiplot, a comprehensive web service for biomedical data analysis and visualization. STRING network analysis was performed based on STRING database.[3] Using all identified interacting proteins of Trx1 or SELM as input, protein interactions were filtered with STRING interaction confidence > 0.7. Statistical analysis of the interaction network was performed based on the interactions in the networks compared to randomly expected frequency of interactions. Network visualization was performed using Cytoscape software version 3.10.0. The MCODE plugin of Cytoscape was used to extract the interconnected modules under default settings for other parameters.

Co-immunoprecipitation of SELM and interaction proteins

HEK 293T cells were cultured in 6 well-cell culture dishes until 80% cell confluency. The plasmids expressing full length Strep tagged SELM-U48C and Flag tagged crosslinked proteins (HSPA5, PRDX4, PLGT3, CALR) were co-transfected or separately transfected. After 48 hours, cells were collected, lysed and immunoprecipitated by 10 μl prewashed Flag beads or Streptactin Beads 4FF.

Beads were resuspended in 15 μl 1×SDS loading and boiled at 95℃ for 5 minutes. The supernatant was analyzed by western blots.

***In vitro* disulfide trapping of PRDX4 and SELM**

A 10 μl mixture containing PRDX4 (50 μg/mL) and SELM (50 μg/mL) in HEPES buffer (pH 7.5) was incubated at room temperature for 2 h. 10 ul reaction product was separated by non-reducing SDS-PAGE and analyzed by western blot (Anti- Strep-tag II).

Sequence of eFSYRS for incorporation EFSY in *E. coli*

MDKKPLDVLISATGLWMSRTGTLHKIKHYEISRSKIYIEMACGDHLVVNNSRSCRPARAFRY
HKYRKTCKRCRVSDEDINNFLTRSTEGKTSVKVKVVSEPKVKKAMPKSVSRAPKPLENPVS
AKASTDTSRSVPSPAKSTPNSPVPTSASAPALTKSQTDRLEVLLNPKDEISLNSGKPFRELESE
LLSRRKKDLQQIYAEEREGGGSGGGGGSGGGGGSGGGGQAWGSRPPAAECATQRAPGSVV
ELLGKSYPQDDHSNLTRKVLTRVGRNLHNQQHHPLWLIKERVKEHFYKQYVGRFGTPLFSV
YDNLSPVVTTWQNFDSLLIPADHPSRKKGDNYYLNRTHMLRAHTSAHQWDLLHAGLDAFL
VVGDVYRRDQIDSQHYPIFHQLEAGRLFSKHELFAGIKDGESLQLFEQSSRSAHKQETHTME
AVKLVEFDLKQTLTRLMAHLFGDELEIRWVDCYCPFGHPSFEMEINFHGEWLEVLGCGVME
QQLVNSAGAQDRIGWGFGLGLERLAMILYDIPDIRLFWCEDERFLKQFCVSNINQKVKFQPL
SK

Sequence of eFSYRS for incorporation EFSY in mammalian cell

MDKKPLDVLISATGLWMSRTGTLHKIKHYEISRSKIYIEMACGDHLVVNNSRSCRPARAFRY
HKYRKTCKRCRVSDEDINNFLTRSTEGKTSVKVKVVSEPKVKKAMPKSVSRAPKPLENPVS
AKASTDTSRSVPSPAKSTPNSPVPTSASAPALTKSQTDRLEVLLNPKDEISLNSGKPFRELESE
LLSRRKKDLQQIYAEEREGGGSGGGSGGGSGGGSGGGSQAWGSRPPAAECATQRAPGSVVE
LLGKSYPQDDHSNLTRKVLTRVGRNLHNQQHHPLWLIKERVKEHFYKQYVGRFGTPLFSVY
DNLSPVVTTWQNFDSLLITADHPCRRKGDNYYLNRTHMLRSHTSAHQWDLLHAGLDAFLV

VGDVYRRDQIDSQHYPIFHQLEAGRLFTKHELFAGIKDGESQQLFEQSSRSAHKQESHTMEA
VKLVEFDLKQTLTRLMAHLFGDEPEIRWVDCYCPFGHPSFEMEINFHGEWLEVLGCGVLEQ
QLVNSAGAQDRIGWGFGLGLERLAMILYDIPDIRLFWCEDERFLKQFCVSNINQKVKFQPLS
K

Sequence of tRNA 3C11-chPheT

GTGAGAGTGATCATGTAGATCGAACGGACTCTAAATCCGTTCAGCCGGGTTAGATTCCCG
GCTCTCACACCA

Supplementary References

1. Sherman BT, *et al.* DAVID: a web server for functional enrichment analysis and functional annotation of gene lists (2021 update). *Nucleic Acids Res* **50**, W216-W221 (2022).

2. Huang da W, Sherman BT, Lempicki RA. Systematic and integrative analysis of large gene lists using DAVID bioinformatics resources. *Nature protocols* **4**, 44-57 (2009).

3. Szklarczyk D, *et al.* The STRING database in 2021: customizable protein-protein networks, and functional characterization of user-uploaded gene/measurement sets. *Nucleic Acids Res* **49**, D605-D612 (2021).
