# Supplementary material for: Characterize direct protein interactions with enrichable, cleavable and latent bioreactive unnatural amino acids
Source: Nat Commun. 2024 Jun 18;15:5221. doi: 10.1038/s41467-024-49517-1 (PMC11189575; doi:10.1038/s41467-024-49517-1)
Supplement: Supplementary file 2 — Reporting Summary [file 41467_2024_49517_MOESM2_ESM.pdf]

nature portfolio

Corresponding author(s): Bing Yang

Last updated by author(s): May 22, 2024

Reporting Summary

Nature Portfolio wishes to improve the reproducibility of the work that we publish. This form provides structure for consistency and transparency in reporting. For further information on Nature Portfolio policies, see our Editorial Policies and the Editorial Policy Checklist.

Statistics

For all statistical analyses, confirm that the following items are present in the figure legend, table legend, main text, or Methods section.

n/a	Confirmed	
☐	☒	The exact sample size (*n*) for each experimental group/condition, given as a discrete number and unit of measurement
☐	☒	A statement on whether measurements were taken from distinct samples or whether the same sample was measured repeatedly
☐	☒	The statistical test(s) used AND whether they are one- or two-sided<br>*Only common tests should be described solely by name; describe more complex techniques in the Methods section.*
☒	☐	A description of all covariates tested
☐	☒	A description of any assumptions or corrections, such as tests of normality and adjustment for multiple comparisons
☐	☒	A full description of the statistical parameters including central tendency (e.g. means) or other basic estimates (e.g. regression coefficient) AND variation (e.g. standard deviation) or associated estimates of uncertainty (e.g. confidence intervals)
☐	☒	For null hypothesis testing, the test statistic (e.g. *F*, *t*, *r*) with confidence intervals, effect sizes, degrees of freedom and *P* value noted<br>*Give P values as exact values whenever suitable.*
☒	☐	For Bayesian analysis, information on the choice of priors and Markov chain Monte Carlo settings
☒	☐	For hierarchical and complex designs, identification of the appropriate level for tests and full reporting of outcomes
☒	☐	Estimates of effect sizes (e.g. Cohen's *d*, Pearson's *r*), indicating how they were calculated

Our web collection on statistics for biologists contains articles on many of the points above.

Software and code

Policy information about availability of computer code

Data collection	Thermo Scientific Xcalibur 4.5.445.18 was used to collect the LC-MS/MS data; SCIEX OS 3.1.6.44 used to collect the LC-MS data. Tanon 5200 was used to collect the immunoblots. ImageView 4.11 was used to detect fluorescent images.
Data analysis	pLink 2.3.11, OpenUaa and AixUaa were used for identification of cross-linking peptides; pFind 3.2.0 was used for identification of regular peptides; Graphpad Prism 8.0.2 was used for statistical analysis; PyMOL 2.3.3 was used to visualize of crystal structures of proteins; ChemDraw 19.0 was used to draw structures of molecular; Cytoscape 3.10.0 was used to visualize the interaction networks.<br>AixUaa is developed in Python and is freely available. The latest software version is available at https://github.com/BUAA-LiuLab/AixUaa.

For manuscripts utilizing custom algorithms or software that are central to the research but not yet described in published literature, software must be made available to editors and reviewers. We strongly encourage code deposition in a community repository (e.g. GitHub). See the Nature Portfolio guidelines for submitting code & software for further information.

Data

Policy information about availability of data

All manuscripts must include a data availability statement. This statement should provide the following information, where applicable:

- Accession codes, unique identifiers, or web links for publicly available datasets
- A description of any restrictions on data availability
- For clinical datasets or third party data, please ensure that the statement adheres to our policy

The mass spectrometry data generated in this study have been deposited in the ProteomeXchange Consortium under accession code PXD047084 [https://proteomecentral.proteomexchange.org/cgi/GetDataset?ID=PXD047084]. Source data of box blots in Figure 3a and uncropped versions of gels or blots are provided with this paper. Any Supplementary Information (methods, figures, notes), and Supplementary Data (reagents, DNA sequences and list of cross-linking peptides) are available in this paper.

Research involving human participants, their data, or biological material

Policy information about studies with human participants or human data. See also policy information about sex, gender (identity/presentation), and sexual orientation and race, ethnicity and racism.

Reporting on sex and gender	N/A
Reporting on race, ethnicity, or other socially relevant groupings	N/A
Population characteristics	N/A
Recruitment	N/A
Ethics oversight	N/A

Note that full information on the approval of the study protocol must also be provided in the manuscript.

Field-specific reporting

Please select the one below that is the best fit for your research. If you are not sure, read the appropriate sections before making your selection.

☒ Life sciences ☐ Behavioural & social sciences ☐ Ecological, evolutionary & environmental sciences

For a reference copy of the document with all sections, see nature.com/documents/nr-reporting-summary-flat.pdf

Life sciences study design

All studies must disclose on these points even when the disclosure is negative.

Sample size	Our manuscript is a mass spectrometry methodology study, we analyzed purified protein samples, E. coli samples and HEK293T samples. Each sample was measured with at least 3 technical replicates.
Data exclusions	No data was excluded from our analysis. All attempts to repeat the experiment were successful.
Replication	All mass spectrometry samples were measured at least 3 technical replicates. The experiments in the figure 1b, 1c, 1h, 1i, 2b, 2c, 4a, 4d, 5b, 5c, 5e and 5h were repeated twice with similar results.
Randomization	Randomization was not relevant in this study. Control is not needed. Our research focuses on cross-linking peptide identification, the well-matched spectrum confirms the reliability of our results.
Blinding	Blinding was not relevant here because group allocation was not involved in this study.

Reporting for specific materials, systems and methods

We require information from authors about some types of materials, experimental systems and methods used in many studies. Here, indicate whether each material, system or method listed is relevant to your study. If you are not sure if a list item applies to your research, read the appropriate section before selecting a response.

Materials & experimental systems

n/a	Involved in the study
☐	☒ Antibodies
☐	☒ Eukaryotic cell lines
☒	☐ Palaeontology and archaeology
☒	☐ Animals and other organisms
☒	☐ Clinical data
☒	☐ Dual use research of concern
☒	☐ Plants

Methods

n/a	Involved in the study
☒	☐ ChIP-seq
☐	☒ Flow cytometry
☒	☐ MRI-based neuroimaging

Antibodies

Antibodies used

The following antibodies were used:
anti-His tag, Mouse, Proteintech, cat. no. HRP-66005, 1:10000
anti-FLAG tag, Mouse, Proteintech, cat. no. HRP-66008, 1:10000
anti- Strep-tag II, Mouse, Wuhan Dian Biotechnology, cat. no. 2098, 1:10000
anti-biotin, HRP-linked Antibody, Cell Signaling Technology, cat. no. 7075S, 1:2500
Goat anti-Mouse IgG (H+L) Secondary Antibody, Jackson ImmunoResearch, Code. 115-035-003, 1:10000

Validation

These antibodies have been validated by suppliers, by relevant citations. The manufacturer's websites are provided as follows.
anti-His tag: https://www.ptgcn.com/products/His-Tag-Antibody-66005-1-Ig.htm
anti-FLAG tag: https://www.ptgcn.com/products/Flag-tag-Antibody-66008-4-Ig.htm
anti- Strep-tag II: https://dia-an.com/index/product.html?pro_id=4470&aid=48,49#view_zjy
anti-biotin:https://www.cellsignal.cn/products/secondary-antibodies/anti-biotin-hrp-linked-antibody/7075
Goat anti-Rabbit IgG (H+L) Secondary Antibody:
https://www.jacksonimmuno.com/catalog/products/115-035-003

Eukaryotic cell lines

Policy information about cell lines and Sex and Gender in Research

Cell line source(s)	HEK293T cell was obtained from American Type Culture Collection (ATCC, CRL-3216).
Authentication	Cells were authenticated by STR profiling by vendors.
Mycoplasma contamination	All cells were tested negative for mycoplasma.
Commonly misidentified lines (See ICLAC register)	No commonly misidentified cell lines were used in this study.

Plants

Seed stocks

Report on the source of all seed stocks or other plant material used. If applicable, state the seed stock centre and catalogue number. If plant specimens were collected from the field, describe the collection location, date and sampling procedures.

Novel plant genotypes

Describe the methods by which all novel plant genotypes were produced. This includes those generated by transgenic approaches, gene editing, chemical/radiation-based mutagenesis and hybridization. For transgenic lines, describe the transformation method, the number of independent lines analyzed and the generation upon which experiments were performed. For gene-edited lines, describe the editor used, the endogenous sequence targeted for editing, the targeting guide RNA sequence (if applicable) and how the editor was applied.

Authentication

Describe any authentication procedures for each seed stock used or novel genotype generated. Describe any experiments used to assess the effect of a mutation and, where applicable, how potential secondary effects (e.g. second site T-DNA insertions, mosiacism, off-target gene editing) were examined.

Flow Cytometry

Plots

Confirm that:

- [x] The axis labels state the marker and fluorochrome used (e.g. CD4-FITC).
- [x] The axis scales are clearly visible. Include numbers along axes only for bottom left plot of group (a 'group' is an analysis of identical markers).
- [x] All plots are contour plots with outliers or pseudocolor plots.
- [x] A numerical value for number of cells or percentage (with statistics) is provided.

Methodology

Sample preparation	HEK293T cells transfected with EGFP were analyzed to test the incorporation efficiency of unnatural amino acid. HEK293T cells were cultured in 12-well plate. pNEU-eFSYRS and pRK5M-EGFP(Y151TAG) were co-transfected . Six hours post transfection, the media containing transfection complex were replaced with fresh DMEM media with 10% FBS in the presence or absence of different concentrations of eFSY. After culture at 37°C for 48 h, transfected cells were collected and resuspended in 500ul PBS for analysis.
Instrument	Beckman CytoFlex S
Software	CytoFLEX was used to collect the flow cytometry data; FlowJo was used to analyze the flow cytometry data.
Cell population abundance	20000 cells were collected and analyzed in each sample.
Gating strategy	HEK 293T cells trasfected with EGFP were used to set appropriate forward scatter (FSC) and side scatter (SSC) gains. HEK 293T cells without transfection were used as negative control to set FITC gate.

- [x] Tick this box to confirm that a figure exemplifying the gating strategy is provided in the Supplementary Information.
