# Supplementary material for: Characterize direct protein interactions with enrichable, cleavable and latent bioreactive unnatural amino acids
Source: Nat Commun. 2024 Jun 18;15:5221. doi: 10.1038/s41467-024-49517-1 (PMC11189575; doi:10.1038/s41467-024-49517-1)
Supplement: Supplementary file 3 — Description of Additional Supplementary Files [file 41467_2024_49517_MOESM3_ESM.pdf]

Description of Additional Supplementary Files

File Name: Supplementary Data 1

Description: PCR Primers for plasmid construction.

File Name: Supplementary Data 2

Description: Companies and catalog numbers of commercial reagents.

File Name: Supplementary Data 3

Description: Cross-linked peptides identified in Trx1 and SELM samples.
